# Optimizing Early Compressive Strength of Ultrafine POFA-Based Green HSC: An Experimental and RSM Approach with Silica Fume and Hybrid Fibers

**DOI:** 10.12688/f1000research.175237.2

**Published:** 2026-02-26

**Authors:** Aktham H Alani, Doaa Ahmed, Qasim A. Al-obaidi, Nahla Hilal, Megat Azmi Megat Johari, Hayder Sadeq Al-Aasam

**Affiliations:** 1 Department of Engineering Affairs, University of Fallujah, Al-Fallujah, Al Anbar Governorate, Iraq; 2 University of Bilad AL-Rafidain, Baqubah, Iraq; 3 University of Fallujah, Scientific Affairs Department, Fallujah, Iraq; 4 Universiti Sains Malaysia School of Civil Engineering, Nibong Tebal, Penang, Malaysia; 5 Technical Engineering College, Al-Esraa University, Baghdad, Iraq

**Keywords:** Green concrete; Palm Oil Fuel Ash; Response surface method; Early compressive strength; Hybrid fiber; Steel fiber; PET fiber; Silica fume; Sustainability

## Abstract

Recently, studies have focused on producing green, sustainable concrete with improved environmental performance, utilizing recycled waste materials. The early compressive strength of Green High Strength Concrete (GHSC) containing up to 60% ultrafine palm oil fuel ash (UPOFA) has been optimized utilizing hybrid materials, Silica Fume (SF), steel fiber (ST), and Polyethylene Terephthalate (PET) fiber. UPOFA was used as a substitute binder for cement in GHSC manufacture at 0%, 30%, and 60% replacement levels. SF was substituted for 0%, 10%, and 20% of the residual cement mass. Steel fiber and PET fiber were included at a rate of 1% of the total binder mass. The mix design parameters were optimized using the Response Surface Method (RSM) with a central composite design (CCD) approach. The experimental results showed that the GHSC containing 30% UPOFA, 20% SF, 1% ST, and 1% PET fiber reached greater strength at 3, 7, and 28 days. The incorporation of 20% SF, 1% ST, and 1% PET fibers enhanced the early ages strength reduction of 60% UPOFA-GHSC by 17%, 25%, and 18% at 3, 7, and 28 days, respectively, relative to the control mix. The constructed models showed significant correlation values (R ^2^) of 0.9697, 0.9546, and 0.9674 for compressive strength at 3, 7, and 28 days, respectively. Hence, RSM may be an efficient technique for improving mix design while lowering environmental impact by limiting waste volume and energy usage in cement manufacture, which contributes to the sustainability of concrete.

## 1. Introduction

The construction industry currently heavily relies on concrete, consuming around 10 billion tons annually. This demand is projected to rise significantly by 2050, potentially reaching 18 billion tons per year due to global population growth. However, this substantial production of concrete also results in a significant generation and emission of CO _2_, posing a major environmental concern. ^1, 2^ This issue requires further concern for developing eco-efficient and durable concrete alternatives. Numerous studies have shown that the cement sector alone contributes to about 8% of global CO _2_ emissions, underscoring the importance of collective responsibility. ^3– 6^ Researchers have played a pivotal role in examining the viability of using waste materials with significant pozzolanic potential as supplementary cementitious components in concrete formulations. ^7, 8^ Their efforts have been part of a larger initiative to identify industrial and agricultural wastes that might serve as substitutes for cement and aggregates, thereby mitigating the harmful effects associated with cement production. ^9, 10^ A common solution to this issue is replacing cement with recycled waste materials that have similar cementitious properties or pozzolanic attributes. Recent studies have identified palm oil fuel ash (POFA) as an effective pozzolanic material for cement replacement, highlighting its cost-effectiveness and environmental sustainability. ^11, 12^ POFA is a by-product derived from the combustion of wastes generated from palm oil extraction, including empty fruit bunches, palm kernel shells, and fibers. So, POFA is the final residue after the combustion of these wastes to heat up the boiler to generate electricity in the palm oil manufacturing process. ^13, 14^ Grinding–burning–regrinding is a novel treatment procedure that has been recently implemented to enhance the pozzolanic reactivity of the original POFA material and produce ultrafine POFA (UPOFA). ^8^ Several studies have suggested that UPOFA can be used as an effective pozzolanic supplement binder replacing significant volume of Portland cement, leading to a significant improvement in the engineering properties of concrete and ensuring superior resistance to aggressive ions from the exposure environment. ^15, 16^ Nonetheless, several drawbacks have been reported from the substantial use of UPOFA as a supplementary cementitious ingredient in green concrete. These include significant reduction in early strength of concrete, where up to a 40% reduction in early concrete strength has been observed. Besides, the tensile strength reduced with UPOFA inclusion up to 20% content. This decrease is primarily due to the reduction in the cement binder content that the UPOFA replaced. These findings have major and crucial practical ramifications for the building sector, as demonstrated by numerous previous studies. ^15, 17, 18^ Moreover, the replacement rates of HSGC are probably high in POFA, which is constrained in the areas of the application where the removal of the formwork or pre-stressing of the structures is required in a very short period of time, and the high early-age strengths are required. ^16^

As a result, studies revealed significant findings about the use of silica fume (SF), a common pozzolanic additive, in high-strength concrete mixes. It was found that silica fume can effectively accelerate the development of early concrete strength by increasing cement hydration via the huge surface area of the much finer silica fume particles relative to cement. The very fine particle of silica fume, which translates into an enormous surface area, not only enhances its filler effect but also accelerates the cement hydration as well as hastens the onset of pozzolanic reaction, leading to a substantial increase in the compressive strength of concrete at a young age. ^19– 21^ However, pozzolanic cementitious composite materials are prone to cracking due to their brittle characteristics. ^15, 22, 23^ Therefore, the incorporation of fibers to produce what is known as fiber-reinforced concrete was considered to address the issues of brittleness and reduction in compressive and tensile strength with UPOFA inclusion.

There have been several attempts to increase concrete’s strength and durability performance. ^24, 25^ Researchers discovered that by incorporating appropriate fibers into the concrete matrix, they could increase the mechanical characteristics of the material and improve its strength properties. These properties prevented micro-cracks from expanding into macro-cracks, which improved the material’s overall durability. There were reports of effective incorporation of various fibers, such as steel fiber, polyvinyl alcohol, polypropylene, basalt, glass, and carbon fiber, etc. ^26– 28^ Steel fiber is the most common reinforcement used in concrete matrices, efficiently bridging micro-cracks caused by load conditions across crack surfaces, preventing macro-fracture growth. Steel fiber concentrations of less than 1.0% significantly increase concrete impact resistance. The studies attributed that to the steel fibers, which inhibit micro-crack propagation and their transition to macro-cracks during the impact compression process. ^29^ Mezzal et al. ^30^ investigated the effect of adding steel fiber at a volumetric percentage of 1% on concrete’s impact resistance, flexural, and tensile strength.

The fracturing of concrete is a progressive, multi-scale phenomenon that has led to significant interest in incorporating steel fibers alongside other fibers. This attraction is due to their potential advantages at various loading stages and scales compared to single-fiber reinforcements. ^31, 32^ In addition, the use of steel fibers in concrete elevates the structural weight of the material. The workability of the mix diminishes owing to the balling effect as the percentage of steel fibers increases. Steel fiber-reinforced concrete contains electric and magnetic fields, and the steel fibers inside the concrete are susceptible to corrosion. Nevertheless, fiber-reinforced concrete using a single fiber type offers reinforcement only at one level and within a restricted range of crack apertures. The appropriate mixture ratio of steel and synthetic fibers may provide excellent concrete performance, beyond the cumulative efficacy of the individual fibers. ^32– 34^ Recently, the recycling of single-use plastic bottles and containers has emerged as a worldwide concern due to the proliferation of plastic products. Polyethylene terephthalate (PET) is a key material in a variety of drinking containers and other consumer products. Its disposal is challenging and detrimental to the environment. However, there is reassurance in the ongoing efforts of researchers investigating PET waste as reinforcement in concrete, offering a potential method for recycling. ^35, 36^ Recent research found that inserting 0.25%-2% PET fibers into a concrete mix increased its strength by 43.4% at 28 days. ^37^ The addition of 1% plastic fibers increased the flexural and tensile strengths of concrete approximately by 17 % and 15 %, respectively. ^38– 40^ Alani et al. ^41^ discovered that adding 1% PET to ultra-high-strength concrete containing 20% SF and 25% UPOFA increased compressive strength by 18%, 21%, and 27% at 3, 7, and 28 days, respectively, as compared to the plain concrete mix. Despite these positive results for UPOFA in green concrete manufacturing, the majority of current research demonstrates that its use in concrete remains limited by reducing concrete strength at early ages and requires further investigation. However, somehow these studies suggested the limitation of the POFA used because the highest was limited. The compressive strength was determined at a POFA replacement level of 20%, and the ultimate highest compressive strength of 28-days compressive strength achieved with the concretes that used POFA ranged between 60 and 86 MPa. ^17, 18^ Hereby, the effect of the combination of SF, steel, and recycled PET fibers on GHSC strength containing up to 60% UPOFA has been investigated. The research also aimed to reduce the use of cement, which is linked to the reduction of building costs and the mitigation of the negative environmental impacts of CO _2_ emissions.

### 1.1 Research significance

Researchers are concerned about the early strength loss of green high-strength concrete with a high volume of UPOFA replacement. The majority of the studies have looked at how steel or PET fiber affects the compressive and tensile strengths of GHSC. Recent innovative research has employed fibers to investigate early GHSC strength behavior at UPOFA concentrations as low as 40% of the total binder.

This study aimed to investigate the coupling effects of steel, PET fibers, and SF on the compressive and tensile strength of HSGC with low cement content. This includes up to 60% of UPOFA as a replacement binder with cement at the early ages of 3, 7, and 28 days. While UPOFA was employed as a replacement binder at a rate of 30% and 60%, SF was substituted at 10% and 20% of the total mass of the cement binder. Steel fiber was added at 1%, while PET fibers were added at 1%. The compressive and tensile strengths were analyzed and tested. Meanwhile, the response surface methodology (RSM) approach was employed to empirically determine the optimal hybrid fiber and pozzolanic secondary binders to achieve superior strength behavior for GHSC. Thus, the optimum values of UPOFA, SF, steel, and PET fibers for achieving GHSC’s superior compressive strength at early ages have been identified.

## 2. Materials and methods

### 2.1 Cement, UPOFA, and SF

**Figure 1.**
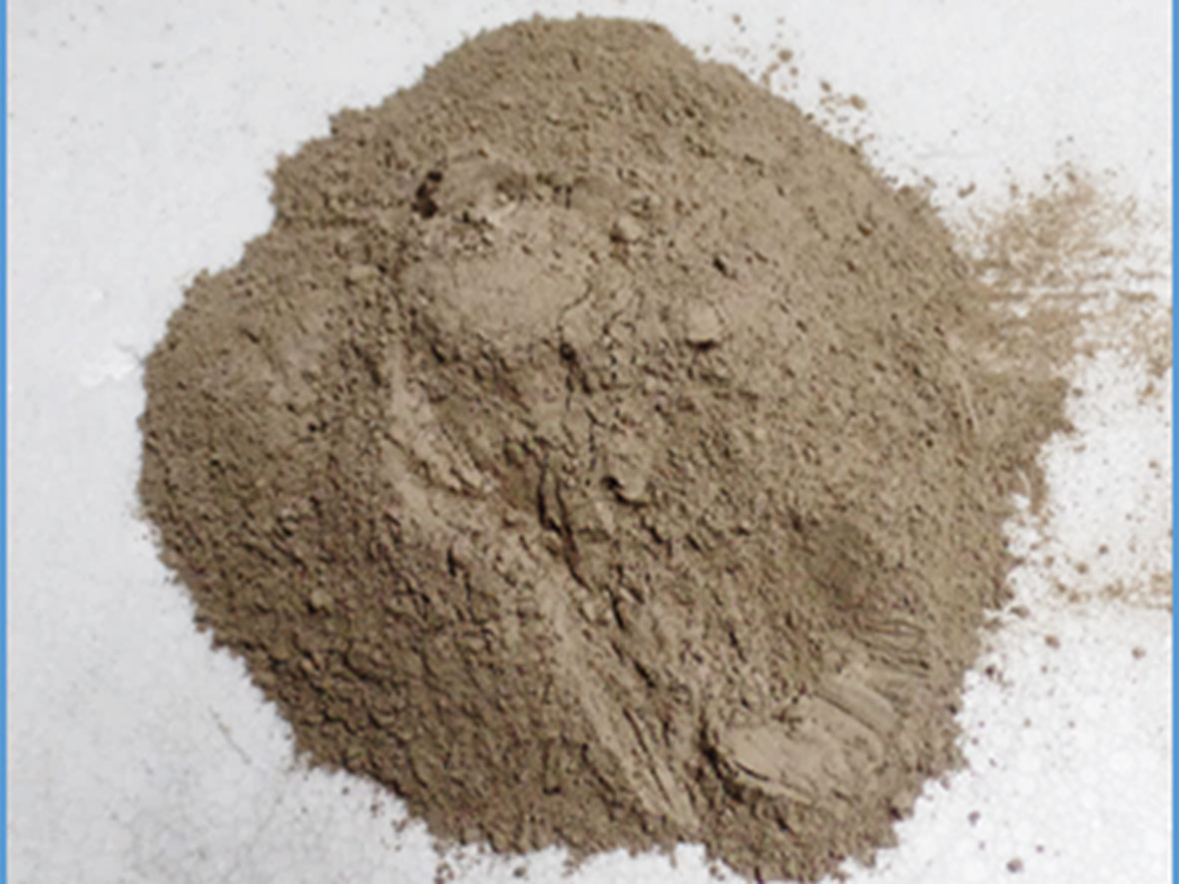
UPOFA.

The main binder is standard Portland cement (OPC) [Type 1, 42.5 R], the specific surface area is 324 m 2/kg, and the specific gravity is 3.15. It is in accordance with the ASTM C150 standards. ^42^ UPOFA ( Figure 1) was selected as a partial replacement binder for its excellent pozzolanic properties and used in concentrations of 30% and 60% of the total mass of the OPC. Similarly, SF was chosen because of its peculiarities, accelerating influence on OPC hydration, and its remarkable pozzolanic activity. SF were substituted by 10% and 20% of the remaining of OPC. Table 1 presents the chemical and physical properties of the binders. Besides these properties, the role and anticipated impact of each material in the performance of the concrete are summarized in Table 2.

**Table 1.** Chemical and physical properties of binder materials.

Property	OPC	UPOFA	SF
SiO _2_ (%)	21.25	64.81	92.25
Al _2_O _3_ (%)	5.11	5.66	0.88
Fe _2_O _3_ (%)	3.35	4.73	1.86
CaO (%)	61.82	8.24	0.94
MgO (%)	2.91	4.63	0.96
K _2_O (%)	0.38	6.37	1.37
SO _3_ (%)	2.42	0.36	0.35
Na _2_O (%)	0.26	0.063	-
Loss on ignition (LOI)	2.51	2.55	4.96
Specific gravity	3.15	2.56	2.53
Particle size (μm)	15	2.11	0.1 to 1
Specific surface area (m ^2^/g)	0.324	1.8	2.16

### 2.2 Aggregates

The selected fine aggregate, river sand, has a maximal particle size of 4.75 mm, a specific gravity of 2.67, and a fineness modulus of 2.88. The crushed gravel, which was chosen for its potential as a coarse aggregate, has a specific gravity of 2.71 and a maximum size of 12.5 mm, to ensure that the compressive force may be imposed upon the matrix rather than on a rigid skeleton of aggregates, which decreases the stresses developed at the paste-aggregate interface. The more stress being transmitted by the aggregates and the surrounding matrix in HSC increased the opportunity for homogenous stress distribution. ^43^

### 2.3 Superplasticizer and water

In order to achieve uniform dispersion and improve flowability with a low water-to-binder (w/b) ratio while maintaining high strength, a polycarboxylic ether-based high-range water-reducing admixture was utilized as a superplasticizer, which has a specific gravity of 1.080 and conforms to ASTM C 494 ^44^ specifications for chemical admixtures used in concrete. The concrete mixes and specimen curing in this study were performed with ordinary tap water.

### 2.4 Steel fiber and PET fiber

**Figure 2.**
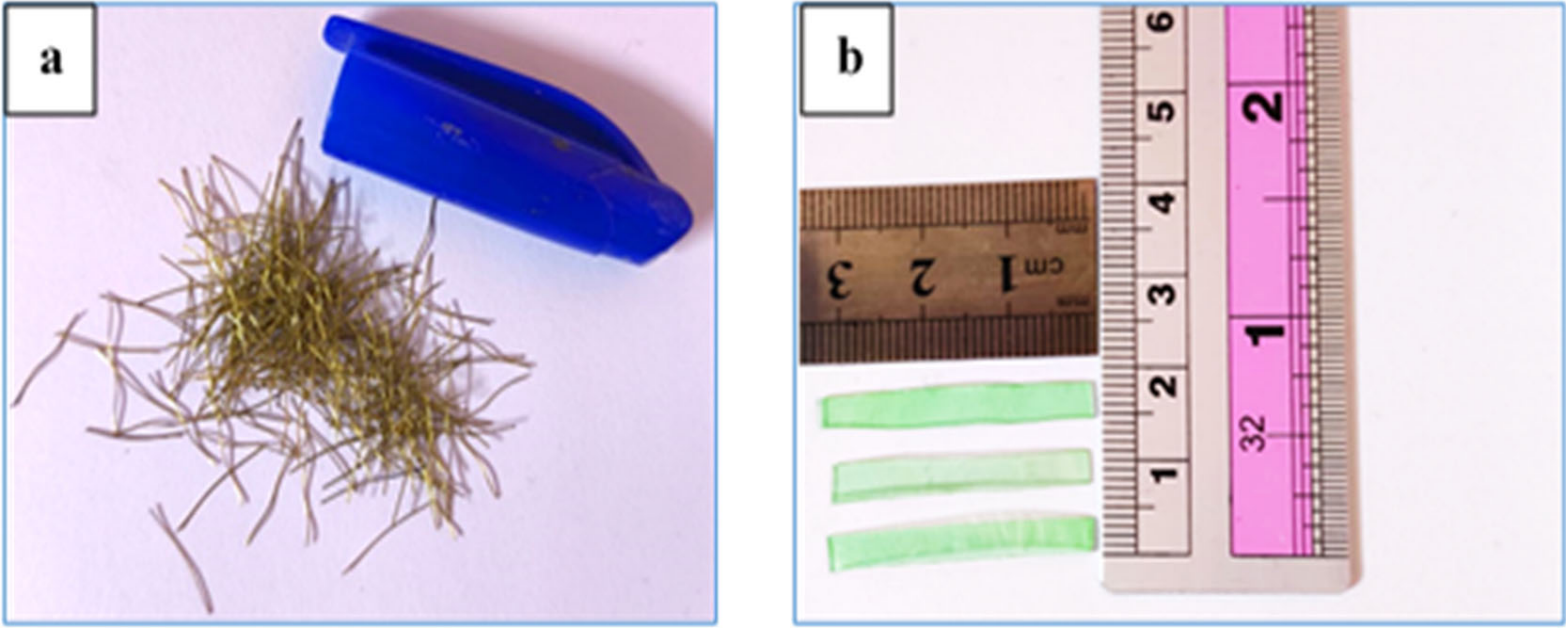
(a) Steel fiber and (b) PET fiber.

Short brass-coated micro-steel fibers used in this study, as shown in Figure 2(a) have a length of 13 mm, a diameter of 0.2 mm, a tensile strength of up to 2600 MPa, and a density of approximately 7.80 g/cm. Plastic waste from polyethylene terephthalate (PET), as shown in Figure 2(b), with the following specifications: average length of 30 mm, breadth of 3 mm, thickness of 0.3 mm, specific gravity of 1.36, and water absorption of 0.17% was used as fiber reinforcement in this study. The contents of the steel and PET fibers were set to 1% volume, which is consistent with previous studies reporting that this amount has improved mechanical performance, crack control, and ductility whilst maintaining workability. ^21, 36^

### 2.5 Experimental design based on RSM approach

The Design-Expert v13 program was employed for modeling, statistical analysis, and response optimization in this study. The central composite design (CCD) is a common method used in RSM. CCD technique is favored to the full factorial and three-level design as it estimates the linear, quadratic and interaction effects with minimum tests, which will lead to a good model fitting, greater accuracy in prediction, reduced amount of residual error, and a high value of R ^2^, that could permit the creation of three-dimensional response surfaces with great reliability. Consequently, it has commonly been considered as the best experimental design to investigate the second-order response models. ^4, 15^ This approach was utilized to establish the relationship between the compressive strength responses at 3, 7, and 28 days and the parameters of UPOFA, SF, steel fiber, and PET fiber. The factorial experimental design for UPOFA and SF included three levels, while the designs for steel and PET fibers comprised two levels. As a result, the RSM technique was employed to optimize the responses of GHSC by varying the levels of these components. The independent variables and their corresponding coding values are shown in Table 3. Besides, Tables 4 and 5 present the results of the CCD approach in variables (%) and mixtures in (kg/m ^3^), respectively, which were implemented in the design of forty-one experimental runs. The CCD design incorporated the center point of the experiment, which was replicated in five runs for M16, in addition to the initial thirty-six trials. This approach aimed to improve the precision of both the experimental design and the analysis. The optimum predictor quadratic model, as described in Equation (1), was used to ascertain the optimal parameters for compressive strength responses at 3, 7, and 28 days.

$$Y=\beta^{0}+\sum_{i=1}^{m}\beta_{\mathrm{ii}}\;X_{i}+\sum_{i=1}^{m}\beta_{\mathrm{ii}}\;X_{i}^{2}+\sum_{i=1}^{m-1}\;\sum_{j}^{m}\beta_{\mathrm{ij}}\;X_{i}X_{j}+e$$ (1)

**Table 2.** Binders role in concrete, proportion%, and expected performance.

Material	Role in concrete	% Proportion	Predictable effect on performance
UPOFA	Partial replacement with cement	30 and 60	Enhances long-term strength, reduces heat of hydration, and reduces early strength.
SF	Partial replacement with cement	10 and 20	Enhance compressive strength at early ages and durability. Refines pore structure and increases brittleness.
Steel fiber	Reinforcement	1	Improve the mechanical properties of concrete at early ages via crack resistance and improve concrete ductility.
PET fiber	Waste fiber reinforcement	1	Improves mechanical properties of concrete and reduce micro crack generation.

**Table 3.** Independent variables, coding, and units.

Factors	Unit	Level		
UPOFA (A)	%	-1	0	1
	%	0	30	60
SF (B)	%	-1	0	1
	%	0	10	20
Steel fiber (C)	%	-1	1	
	%	0	1	
PET fiber (D)	%	-1	1	
	%	0	1	

**Table 4.** CCD mix variables (%) using RSM.

	Mix no.	Mix ID	Variables (%)			
			UPOFA	SF	Steel fiber	PET fiber
GI	M0*	HSC	0	0	0	0
	M1	HSC-ST1	0	0	1	0
	M2	HSC-PET1	0	0	0	1
	M3	HSC-ST1-PET1	0	0	1	1
	M4	HSC-SF10	0	10	0	0
	M 5	HSC-SF10-ST1	0	10	1	0
	M 6	HSC-SF10-PET1	0	10	0	1
	M7	HSC-SF10-ST1-PET1	0	10	1	1
	M8	HSC-SF20	0	20	0	0
	M9	HSC-SF20-ST1	0	20	1	0
	M10	HSC-SF20-PET1	0	20	0	1
	M11	HSC-SF20-ST1-PET1	0	20	1	1
GII	M12	GHSC-U30	30	0	0	0
	M13	GHSC-U30-ST1	30	0	1	0
	M14	GHSC-U30-PET1	30	0	0	1
	M15	GHSC-U30-ST1-PET1	30	0	1	1
	M(16-20)	GHSC-U30-SF10	30	10	0	0
	M21	GHSC-U30-SF10-ST1	30	10	1	0
	M22	GHSC-U30-SF10-PET1	30	10	0	1
	M23	GHSC-U30-SF10-ST1-PET1	30	10	1	1
	M24	GHSC-U30-SF20	30	20	0	0
	M25	GHSC-U30-SF20-ST1	30	20	1	0
	M26	GHSC-U30-SF20-PET1	30	20	0	1
	M27	GHSC-U30-SF20-ST1-PET1	30	20	1	1
GIII	M28	GHSC-U60	60	0	0	0
	M29	GHSC-U60-ST1	60	0	1	0
	M30	GHSC-U60-PET1	60	0	0	1
	M31	GHSC-U60-ST1-PET1	60	0	1	1
	M32	GHSC-U60-SF10	60	10	0	0
	M33	GHSC-U60-SF10-ST1	60	10	1	0
	M34	GHSC-U60-SF10-PET1	60	10	0	1
	M35	GHSC-U60-SF10-ST1-PET1	60	10	1	1
	M36	GHSC-U60-SF20	60	20	0	0
	M37	GHSC-U60-SF20-ST1	60	20	1	0
	M38	GHSC-U60-SF20-PET1	60	20	0	1
	M39	GHSC-U60-SF20-ST1-PET1	60	20	1	1

[M0*] refers to control mix; [U30 and U60] refers to UPOFA30% and 60%; [ST] refer to steel fiber.

Where *Y* represents the response (compressive strength), and *β* and *i* denote the regression coefficients and linear coefficients, respectively. *j* represents the quadratic coefficient, *X _i_X _j_* denotes the coded values for UPOFA, SF, steel fiber, and PET fiber. m signifies the number of components, and e indicates the random error. Except for the control mix, Table 5 showed that OPC weight changed with UPOFA and SF replacement percentages. All GHSC mixes contain the same weight of the GHSC components, which include river sand, crushed gravel, w/b ratio, and SP. This research investigated the effects of UPOFA and SF replacement levels, both with and without steel and PET fibers, on compressive strength responses at 3, 7, and 28 days using analysis of variance (ANOVA). The reliability of the quadratic prediction model was tested using this technique. The model consequently computed a 95% confidence level (P-value) to ascertain statistical significance. Furthermore, the R ^2^ coefficient was calculated after a t-test with a 0.05 threshold of significance. The required model was developed based on actual findings from this research. Diagnostic diagrams were created to evaluate the model’s accuracy.

### 2.6 Samples preparation

HSC and GHSC with and without fibers were prepared using a pan-type concrete mixer. During casting, the samples were consolidated on a vibrating table to obtain maximum density. After a critical 24-hour period, the specimens were carefully removed from their molds and underwent a rigorous water-curing procedure at a constant temperature of 27 ± 2°C until the day of testing. HSC is common for its varying mixture compositions, which provide high-performance characteristics with compressive strength ranging from 62 to 138 MPa.

### 2.7 Test procedures

To attain the primary aim of this research, further experiments were conducted for each mixture of HSC and GHSC as given in Table 5.

**2.7.1 Compressive strength test**

In line with BS EN 12390-3, ^45^ 100 mm concrete cubes were tested using a 2000 kN concrete compression machine at a loading rate of 0.30 MPa/s. The compressive strength was next determined. At least three samples were tested for the different mixtures for each age of 3, 7, and 28 days.

## 3. Experimental results and discussions

The CCD related to varying variable content divides the HSC and GHSC mixtures, both with and without fiber, into three groups, as shown in Tables 4 and 5. The first group (G I) consists of HSC mixes numbered 0–11. The second group (G II) comprises GHSC-U30 mixes numbered 12 through 27. The third group (G III) is known as GHSC-U60 and includes mixes numbered 28 to 39.

### 3.1 Compressive strength

**Figure 3.**
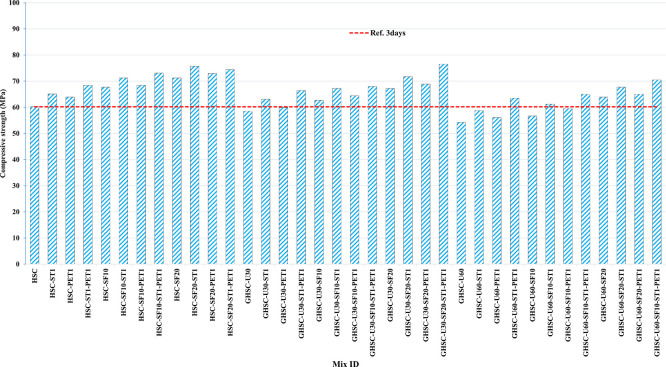
Experimental compressive strength results of GHSC at 3 days.

**Figure 4.**
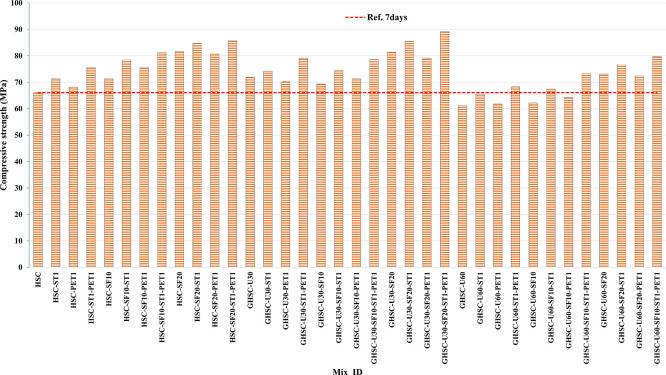
Experimental compressive strength results of GHSC at 7 days.

**Figure 5.**
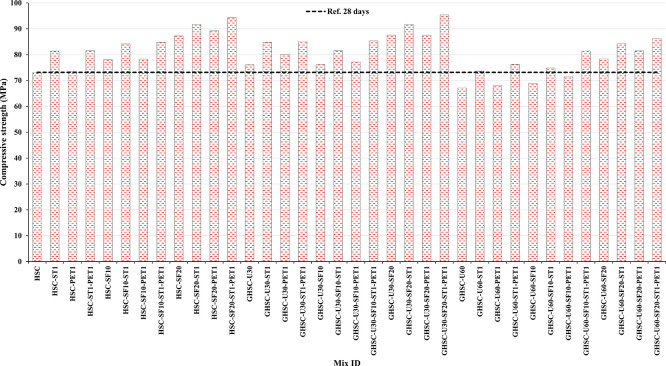
Experimental compressive strength results of GHSC at 28 days.

**Figure 6.**
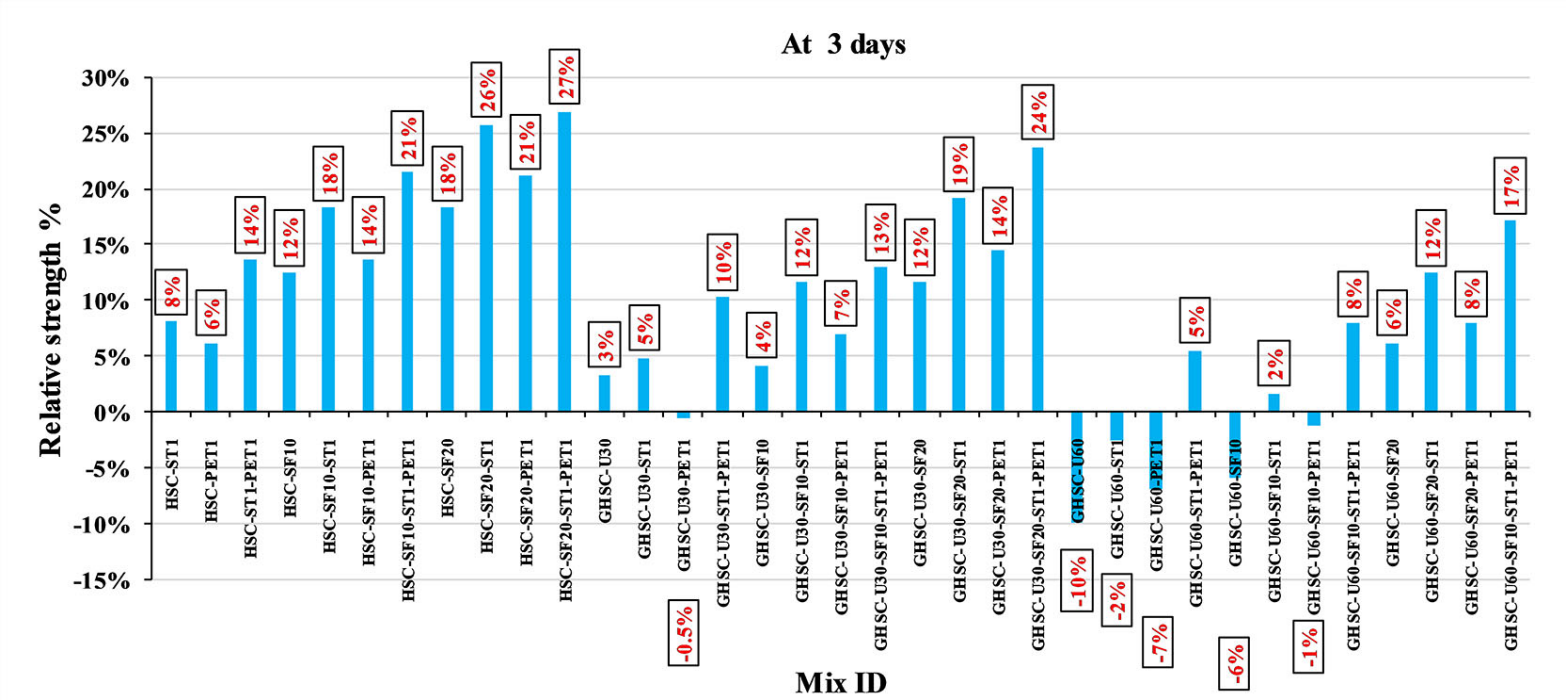
Effect of hybrid materials on the compressive strength of GHSC at 3 days (HSC as reference).

**Figure 7.**
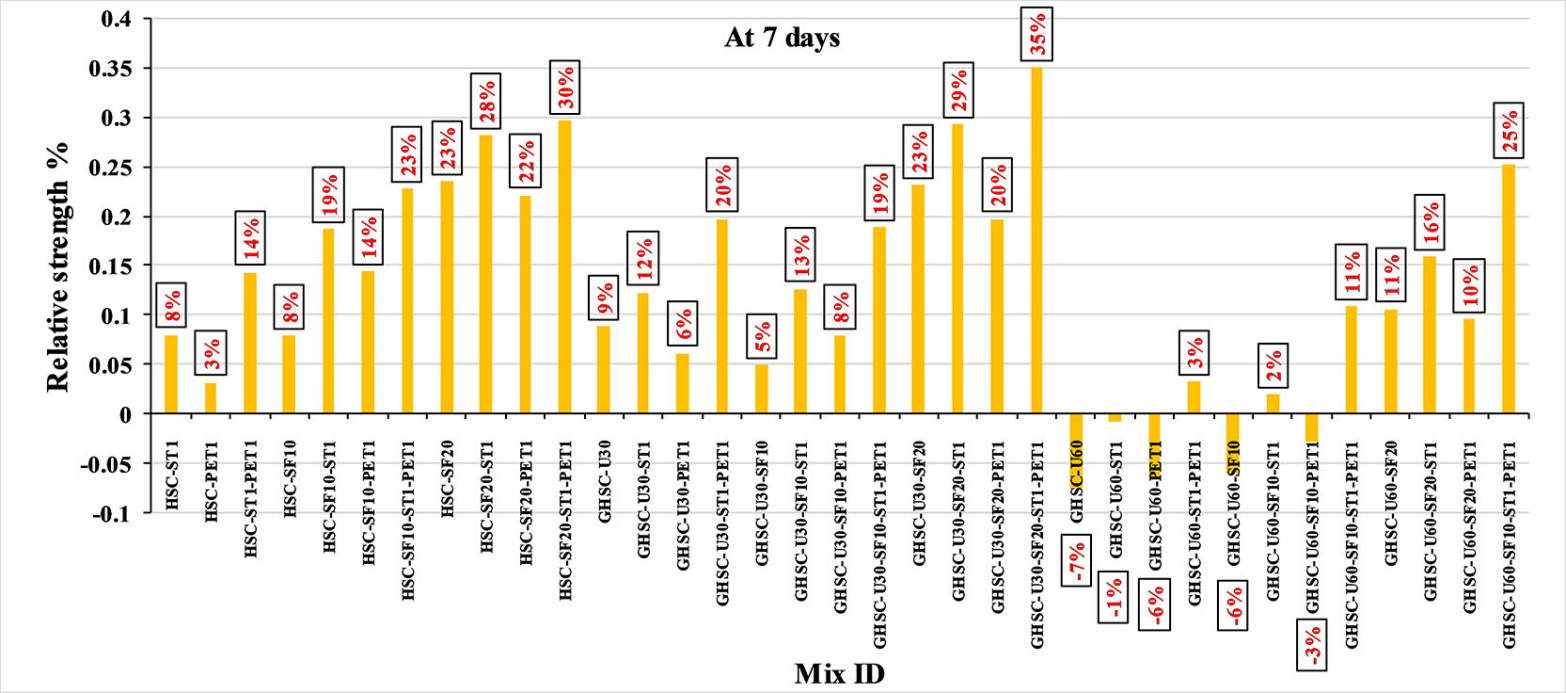
Effect of hybrid materials on the compressive strength of GHSC at 7 days (HSC as reference).

**Figure 8.**
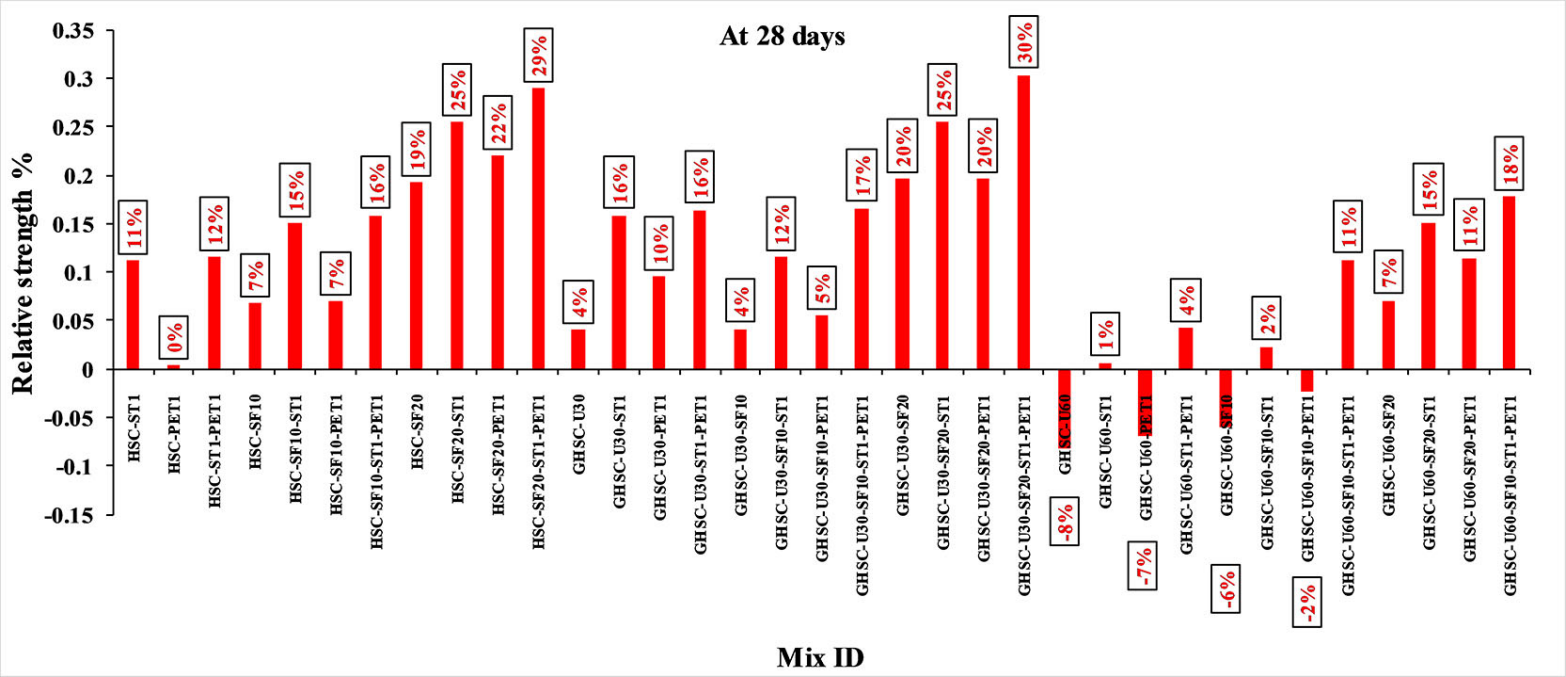
Effect of hybrid materials on the compressive strength of GHSC at 28 days (HSC as reference).

Figures 3, 4, and 5 show that the incorporation of UPOFA reduces the compressive strength of GHSC at all ages (3, 7, and 28 days), with a more significant drop in strength at a higher UPOFA replacement level of U60%. The greater reduction in compressive strength was recorded by M28 (GHSC-U60), about 10%, 7% and 8% relative to the control mix, at 3, 7, and 28 days, respectively, as shown in Figures 6, 7, and 8. This decrease is due to the dilution effect from the low OPC content, contributing towards delayed hydration and pozzolanic reaction of the U60-GHSC mixture, in particular at the early age, which is characterized by a high level of 60% UPOFA. ^46^ As a consequence, the reduced strength was observed from the delayed and lower C-S-H production. Furthermore, the interaction between calcium hydroxide (CH) and silica concentration in POFA influences the concrete’s compressive strength. ^47, 48^ Further, the reduced cement content may minimize the amount of CH released during cement hydration. Thus, mixes with high UPOFA concentrations had lower compressive strength than concrete with a lower POFA fraction. Furthermore, the 75.2% concentration of pozzolanic characteristics (SiO _2_, Al _2_O _3_, and Fe _2_O _3_) may result in high calcium hydroxide (CH) consumption during hydration, as given in Table 1. These factors may result in a CH deficiency, limiting strength development. ^8, 49, 50^ In contrast, the presence of SF, steel fiber, and PET fiber develops compressive strength at all ages of 3, 7, and 28 days.

The compressive strength of HSC and GHSC is considerably improved upon the incorporation of SF at concentrations of 10% and 20% throughout all curing periods. Acceleration in OPC hydration as well as pozzolanic reactions between silica particulate and OPC are responsible for this enhancement. ^51, 52^ The fine SF particles with huge surface area provide sites for the nucleation of OPC hydration products, hence promoting strength gain. In the case of pozzolanic reactions, a curing time of 3 to 4 days is typically required for these reactions (OPC-SF) to begin, according to previous research. ^20, 53^ The compressive strength of GHSC is enhanced over a curing period of 7 to 28 days as a result of the accelerated hydration of conventional OPC and due to the pozzolanic effects of SF. The formation of secondary C-S-H is the consequence of the interaction between (Ca (OH) _2_) produced during cement hydration and SiO _2_ in SF. ^54, 55^ This procedure increases the durability of the concrete by reducing the incidence of microcracks during periods of tension. In the same trend, adding steel and PET fibers improved the compressive strength of HSC and GHSC. The GHSC mixtures with a combination of (UPOFA-SF-ST-PET) registered the highest compressive strength of mixtures in GII and GIII at all curing periods. Hereby, the compressive strength of (M39) GHSC-U60 increased by 17%, 25% and 18% at ages of 3, 7, and 28 days, respectively, related to control mix. This strength development was attributed to the effectiveness of binary binders (UPOFA and SF) in increasing bond formation between steel and PET fibers and other concrete ingredients. The tiny particle size and pozzolanic reactivity of UPOFA and SF reduced void volume and produced extra C-S-H gels, strengthening the aggregate-paste interfacial connection and increasing bonding capabilities with fibers. Adding steel and PET fibers may improve the fracture resistance of fiber-reinforced GHSC composites by limiting micro-crack formation under load, which is accomplished by forming a bridge network within the concrete microstructure. ^31, 41, 56^

Further, particularly in the case of fiber-reinforced concrete (FRC) mixes, the incorporation of supplementary cementitious materials (SCMs) has the potential to enhance the generation of secondary hydration products and be of assistance in the process of filling the pores that are produced at the interface of the fibers. ^57, 58^ A key contribution of SCMs such as SF to improving fiber-matrix interfacial characteristics is that the effectiveness of fibers within a concrete matrix relies heavily on the bond between the fibers and the matrix. Consequently, the load-bearing capacity was enhanced beyond the initial cracking threshold as the randomly dispersed fibers bonded effectively with the matrix, preventing crack propagation and specimen separation. ^59, 60^ In this study, the combination of SF-steel and PET fibers enhanced the compressive strength of UPOFA-GHSC at all ages, even with a high UPOFA content of 60%. The highest strength was observed at M27 (GHSC-U30-SF20-ST1-PET1).

### 3.2 Mathematical modeling and statistical analysis results

The correlation between the variables UPOFA, SF, and steel as well as PET fibers with the compressive strength responses at 3, 7, and 28 days for GHSC mixtures was assessed using the RSM. The model was constructed using actual data. Table 6 summarizes the results of the analysis of variance (ANOVA) conducted on the findings of the quadratic prediction model.

**Table 5.** CCD mix proportion in (kg/m ^3^) using RSM.

	Mix ID		Components (kg/m ^3^)								
			OPC	UPOFA	SF	Steel Fiber	PET Fiber	Fine Agg.	Coarse Agg.	Water	SP
GI	M0*	HSC	570	0	0	0	0	765	1057	155.2	14.25
	M1	HSC-ST1	570	0	0	5.7	0	765	1057	155.2	14.25
	M2	HSC-PET1	570	0	0	0	5.7	765	1057	155.2	14.25
	M3	HSC-ST1-PET1	570	0	0	5.7	5.7	765	1057	155.2	14.25
	M4	HSC-SF10	513	0	57	0	0	765	1057	155.2	14.25
	M 5	HSC-SF10-ST1	513	0	57	5.7	0	765	1057	155.2	14.25
	M 6	HSC-SF10-PET1	513	0	57	0	5.7	765	1057	155.2	14.25
	M7	HSC-SF10-ST1-PET1	513	0	57	5.7	5.7	765	1057	155.2	14.25
	M8	HSC-SF20	456	0	114	0	0	765	1057	155.2	14.25
	M9	HSC-SF20-ST1	456	0	114	5.7	0	765	1057	155.2	14.25
	M10	HSC-SF20-PET1	456	0	114	0	5.7	765	1057	155.2	14.25
	M11	HSC-SF20-ST1-PET1	456	0	114	5.7	5.7	765	1057	155.2	14.25
GII	M12	GHSC-U30	399	171	0	0	0	765	1057	155.2	14.25
	M13	GHSC-U30-ST1	399	171	0	5.7	0	765	1057	155.2	14.25
	M14	GHSC-U30-PET1	399	171	0	0	5.7	765	1057	155.2	14.25
	M15	GHSC-U30-ST1-PET1	399	171	0	5.7	5.7	765	1057	155.2	14.25
	M (16-20)	GHSC-U30-SF10	342	171	57	0	0	765	1057	155.2	14.25
	M21	GHSC-U30-SF10-ST1	342	171	57	5.7	0	765	1057	155.2	14.25
	M22	GHSC-U30-SF10-PET1	342	171	57	0	5.7	765	1057	155.2	14.25
	M23	GHSC-U30-SF10-ST1-PET1	342	171	57	5.7	5.7	765	1057	155.2	14.25
	M24	GHSC-U30-SF20	285	171	114	0	0	765	1057	155.2	14.25
	M25	GHSC-U30-SF20-ST1	285	171	114	5.7	0	765	1057	155.2	14.25
	M26	GHSC-U30-SF20-PET1	285	171	114	0	5.7	765	1057	155.2	14.25
	M27	GHSC-U30-SF20-ST1-PET1	285	171	114	5.7	5.7	765	1057	155.2	14.25
GIII	M28	GHSC-U60	228	342	0	0	0	765	1057	155.2	14.25
	M29	GHSC-U60-ST1	228	342	0	5.7	0	765	1057	155.2	14.25
	M30	GHSC-U60-PET1	228	342	0	0	5.7	765	1057	155.2	14.25
	M31	GHSC-U60-ST1-PET1	228	342	0	5.7	5.7	765	1057	155.2	14.25
	M32	GHSC-U60-SF10	171	342	57	0	0	765	1057	155.2	14.25
	M33	GHSC-U60-SF10-ST1	171	342	57	5.7	0	765	1057	155.2	14.25
	M34	GHSC-U60-SF10-PET1	171	342	57	0	5.7	765	1057	155.2	14.25
	M35	GHSC-U60-SF10-ST1-PET1	171	342	57	5.7	5.7	765	1057	155.2	14.25
	M36	GHSC-U60-SF20	114	342	114	0	0	765	1057	155.2	14.25
	M37	GHSC-U60-SF20-ST1	114	342	114	5.7	0	765	1057	155.2	14.25
	M38	GHSC-U60-SF20-PET1	114	342	114	0	5.7	765	1057	155.2	14.25
	M39	GHSC-U60-SF20-ST1-PET1	114	342	114	5.7	5.7	765	1057	155.2	14.25

Table 6 shows the ANOVA results for the compressive strength parameters that were measured at 3, 7, and 28 days. The models were statistically significant at the 95% confidence level, as the P-values were less than 0.05, as indicated by the results. Furthermore, the substantial P-values for lack of fit (greater than 0.05) throughout each response suggest that the F-value was not significant, indicating a meaningful relationship between the variables and the process responses. Based on empirical correlation, the second-order polynomial illustrates the relationship between compressive strength at 3, 7, and 28 days and the parameters of UPOFA, SF, steel and PET fibers, as shown in the equation below:

Compressive3days=68.22−3.98A+2.52B+2.26C+0.9447D−0.1319AB+0.2342AC+0.2011AD−0.0261BC−0.0845BD+0.3018CD−0.6814A2+0.1798B2 (2)

Compressive 7days=78.67−3.92A+4.39B+3.09C+1.017D−0.1069AB+0.2333AC+0.1392AD−0.0285BC−0.1190BD+0.8339CD−4.13A2+0.7453B2 (3)

Compressive28days=85.36−3.56A+4.83B+3.15C+1.29D−0.2513AB+0.4421AC+0.4196AD−0.0917BC−0.0558BD+0.3881CD−4.48A2+0.9556B2 (4)

Table 7 presents the model validation parameters for all responses. The ANOVA analysis demonstrates a high degree of confidence in the calculation of response efficiencies, represented as R ^2^. The quadratic model demonstrates a commendable fit to the experimental data, as seen by a high R ^2^ value close to one and a suitable correlation with the modified R ^2^. This finding has also been confirmed by Ghafari et al. and Alani et al. ^61, 62^ Further, the coefficient of variation of a model is deemed repeatable if it is less than 10%. ^63^ The repeatability of all models was satisfactory, as demonstrated in Table 7. To establish a favorable accord between the quadratic model and the actual data, the R ^2^ value must be consistent with the adjusted R ^2^. The ANOVA results of this study indicate a highly reliable level of confidence in evaluating response efficacy. The R ^2^ and adjusted R ^2^ values for compressive strength are (R ^2^ = 0.9697 and adjusted R ^2^ = 0.9539) at 3 days, (R ^2^ = 0.9564 and adjusted R ^2^ = 0.9337) at 7 days, and (R ^2^ = 0.9674 and adjusted R ^2^ = 0.9504) at 28 days. Furthermore, the precision values are 32.1, 24.92, and 29.41 at 3, 7, and 28 days, respectively; these values exceed 4, which is considered favorable. Moreover, there exists a substantial level of confidence in the standard deviation, which was much smaller than the mean values obtained. At 3 days, the compressive strength exhibits a standard deviation of 1.19 and a mean of 65.91; at 7 days, the standard deviation is around 1.88 with a mean of 74.26; and at 28 days, the standard deviations are 1.63 and 81.1. This finding implies that the variance analysis was both suitable and statistically significant. A decreasing standard deviation of the created model relative to its mean indicates that the test data exhibit less variability. The experimental data will provide a model with reduced uncertainty. ^10, 64^

**Table 7.** Model validation of compressive strength at 3, 7, and 28 days.

Response	Compressive Strength 3 days	Compressive Strength 7 days	Compressive Strength 28 days
Standard deviation	1.19	1.88	1.63
Mean	65.91	74.26	81.1
R ^2^	0.9697	0.9564	0.9674
Adjusted R ^2^	0.9539	0.9337	0.9504
Predicted R ^2^	0.926	0.8957	0.92451
Precision	32.1	24.92	29.41

**Table 6.** ANOVA results for response surface quadratic model parameters.

Responses	Source	SOS	DF	MS	F-value	p-value	Remark
**Compressive strength 3 days**	**Model**	1045.15	12	87.1	61.40	<0.0001	Sign.
	A-UPOFA	276.29	1	276.29	194.79	<0.0001	Sign.
	B-SF	152.34	1	152.34	107.40	<0.0001	Sign.
	C-ST	134.17	1	134.17	94.59	<0.0001	Sign.
	D-PET	23.37	1	23.37	16.47	0.0005	Sign.
	AB	1.11	1	1.11	0.7846	0.3849	Not- Sign.
	AC	1.32	1	1.32	0.9284	0.3453	Not- Sign.
	AD	0.9703	1	0.9703	0.6841	0.4167	Not- Sign.
	BC	0.0654	1	0.0654	0.0461	0.8319	Not- Sign.
	BD	0.6853	1	0.6853	0.4831	0.4940	Not- Sign.
	CD	3.28	1	3.28	2.31	0.1420	Not- Sign.
	A ^2^	3.71	1	3.71	2.62	0.1193	Not- Sign.
	B ^2^	4.14	1	4.14	2.92	0.1011	Not- Sign.
	C ^2^	0.0000	0				
	D ^2^	0.0000	0				
	Residual	32.62	23	1.42			
	Cor. Total	1077.78	35	87.1			
**Compressive strength 7 days**	**Model**	1775.57	12	147.96	<0.0001	<0.0001	Sign.
	A-UPOFA	268.70	1	268.70	<0.0001	<0.0001	Sign.
	B-SF	462.53	1	462.53	<0.0001	<0.0001	Sign.
	C-ST	249.30	1	249.30	<0.0001	<0.0001	Sign.
	D-PET	26.85	1	26.85	0.0111	0.0111	Sign.
	AB	0.7310	1	0.7310	0.6527	0.6527	Not- Sign.
	AC	1.31	1	1.31	0.5481	0.5481	Not- Sign.
	AD	0.4648	1	0.4648	0.7195	0.7195	Not- Sign.
	BC	0.0782	1	0.0782	0.8828	0.8828	Not- Sign.
	BD	1.36	1	1.36	0.5404	0.5404	Not- Sign.
	CD	25.03	1	25.03	0.0137	0.0137	Sign.
	A ^2^	136.29	1	136.29	<0.0001	<0.0001	Sign.
	B ^2^	71.10	1	71.10	0.0002	0.0002	Sign.
	C ^2^	0.0000	0				
	D ^2^	0.0000	0				
	Residual	80.88	23	3.52			
	Cor. Total	1856.45	35				
**Compressive strength 28 days**	**Model**	1811.68	12	150.97	56.91	<0.0001	Sign.
	A-UPOFA	221.32	1	221.32	83.42	<0.0001	Sign.
	B-SF	559.60	1	559.60	210.93	<0.0001	Sign.
	C-ST	260.57	1	260.57	98.21	<0.0001	Sign.
	D-PET	43.87	1	43.87	16.54	0.0005	Sign.
	AB	4.04	1	4.04	1.52	0.2297	Not- Sign.
	AC	4.69	1	4.69	1.77	0.1967	Not- Sign.
	AD	4.23	1	4.23	1.59	0.2196	Not- Sign.
	BC	0.8067	1	0.8067	0.3041	0.5867	Not- Sign.
	BD	0.2993	1	0.2993	0.1128	0.7400	Not- Sign.
	CD	5.42	1	5.42	2.04	0.1663	Sign.
	A ^2^	160.65	1	160.65	60.55	<0.0001	Sign.
	B ^2^	116.89	1	116.89	44.06	<0.0001	Sign.
	C ^2^	0.0000	0				
	D ^2^	0.0000	0				
	Residual	61.02	23	2.65			
	Cor. Total	1872.70	35				

[SOS]: sum of squares; [DF]: degree of freedom; [MS]: mean square; and [Sign.]: significant.

**Figure 9.**
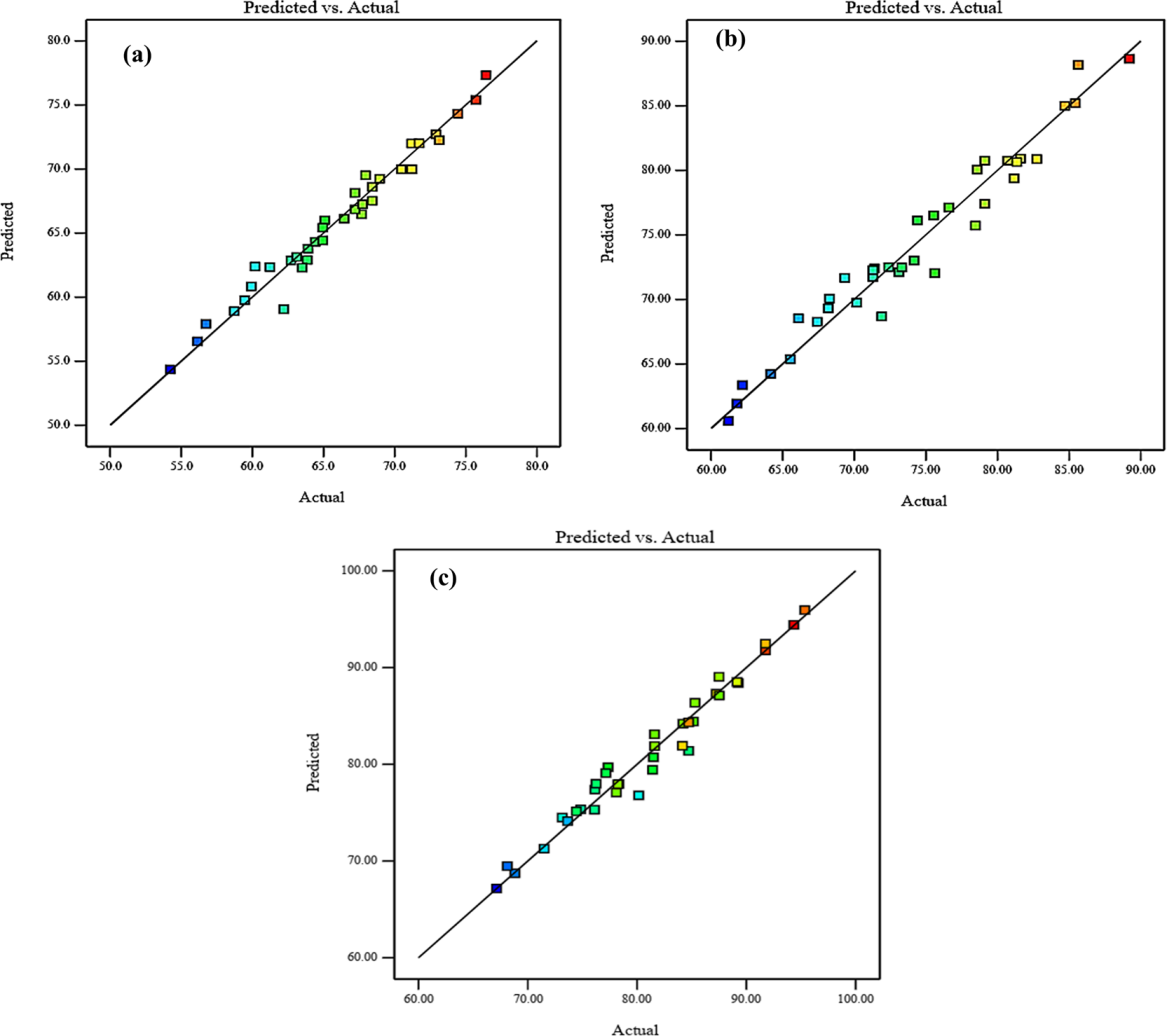
Diagnostics plots, predicted vs actual values for compressive strength at (a) 3 days, (b) 7 days, and (c) 28 days.

**Figure 10.**
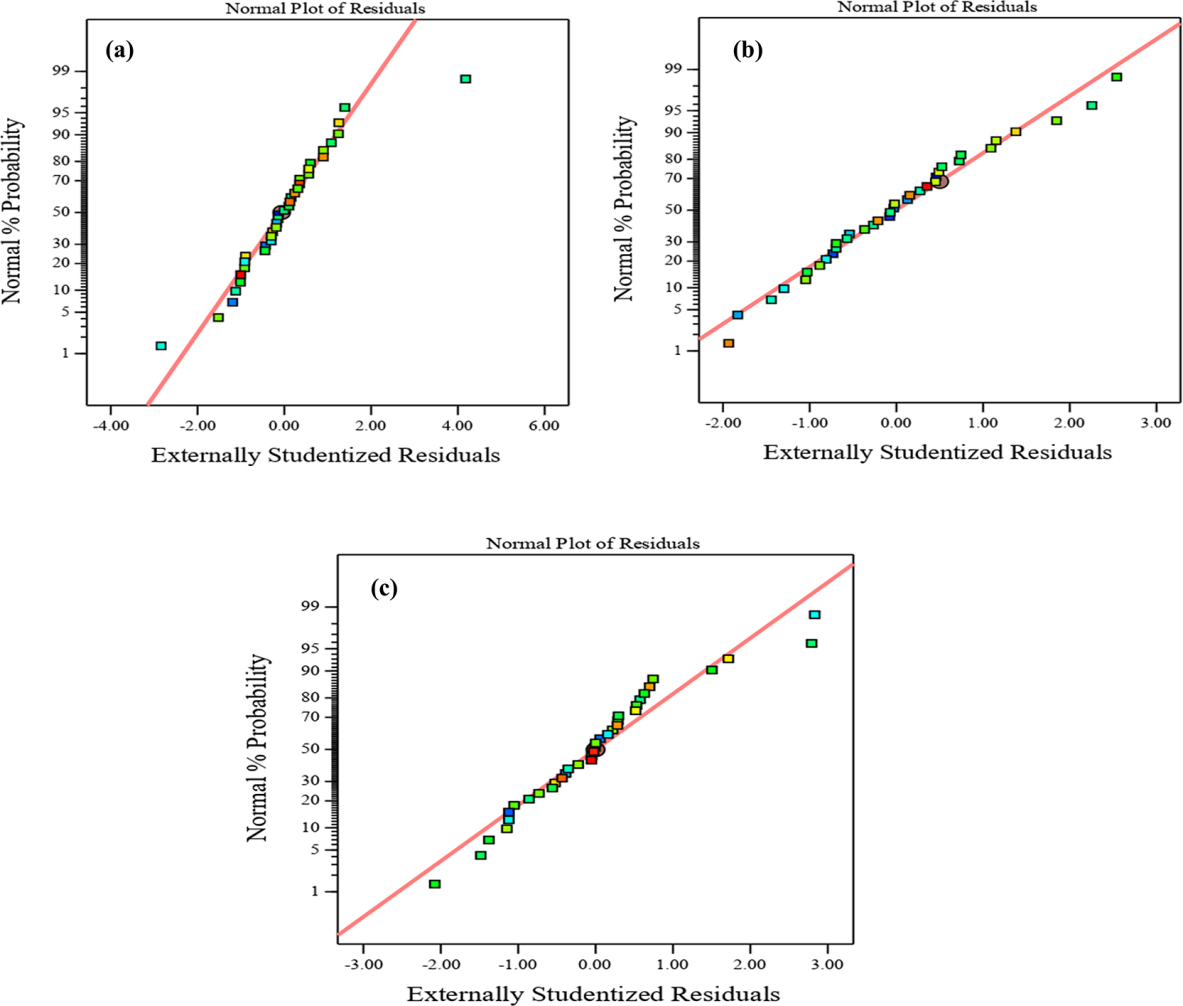
Normal plot of residual for compressive strength at (a) 3 days, (b) 7 days, and (c) 28 days.

Diagnostic charts are illustrated in Figures 9 and 10, which include the normal plot of residuals and a plot comparing expected and actual values. Moreover, the remaining points of the equality line are evenly distributed. The data fell below the line of equality, showing the effectiveness of the regression models. Besides, the scheme of the normal probability plot of all replies is based on the distribution of response model data points. The graphic indicates that the remaining points are nearly linear, which means that the model of the invasion is normally distributed. Thereby, the residuals are randomly spread in a straight line, the distribution of residuals is nearly normal, and the model uniforms the data quite well. These diagnostic plots are instrumental in evaluating the models’ appropriateness and efficacy. ^61^ Thus, Figure 9 and Figure 10, illustrate the plots of the predicted vs. actual values and the normal plots of residuals for compressive strength at 3, 7, and 28 days, respectively. The data indicate a vital correlation between the model-generated compressive strength responses and the real values.

### 3.3 Process analysis

**Figure 11.**
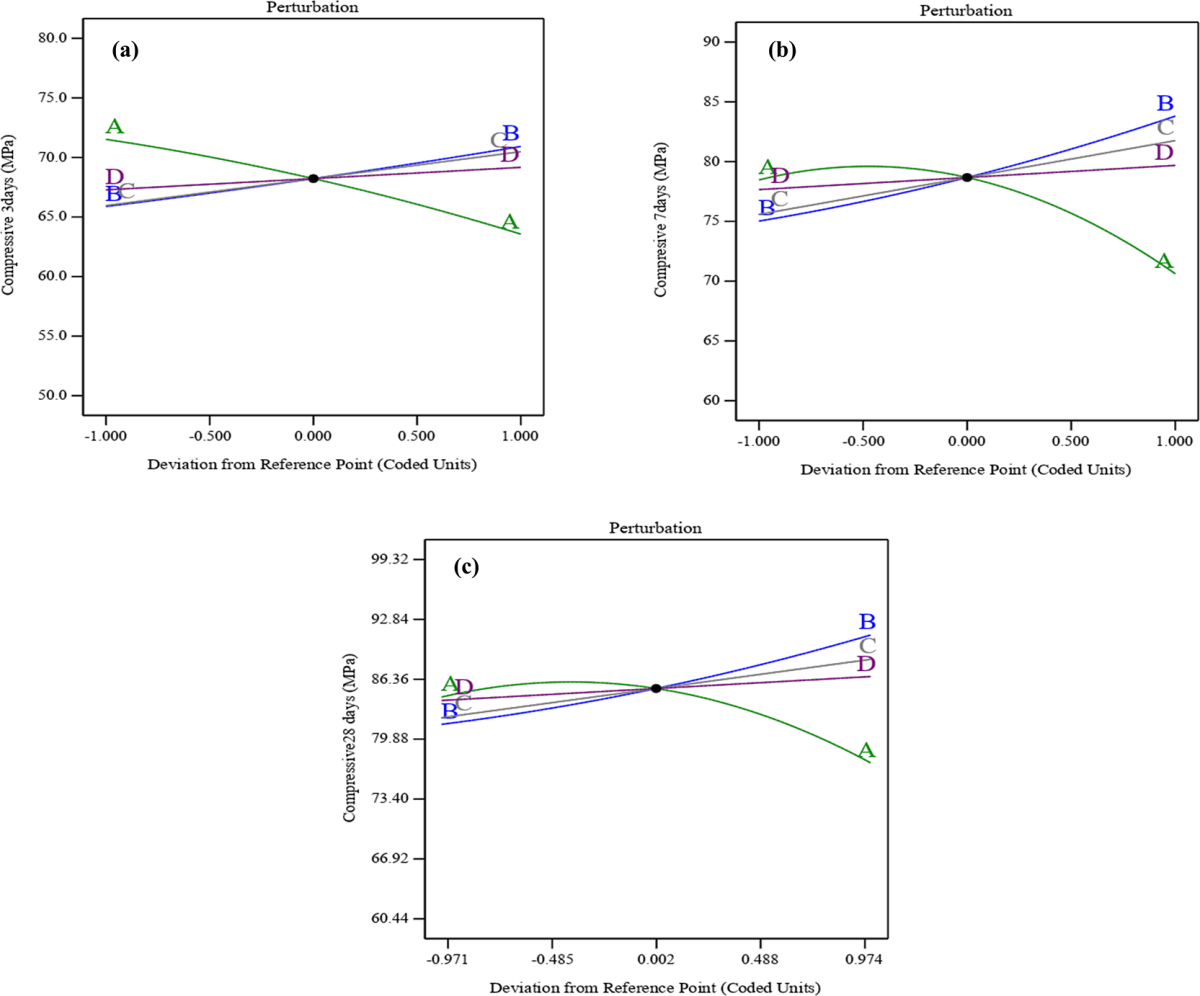
Perturbation plot for compressive strength at (a) 3 days, (b) 7 days, and (c) 28 days.

The perturbation graphs shown in Figures 11 (a), (b), and (c) illustrate how the variables affect compressive strength at early ages of 3, 7, and 28 days. The perturbation plots in the RSM highlight the influence of each parameter by showing how changes in these variables are related to variations in compressive strength. As a result, the combined effect of SF, ST, and PET substantially mitigates the compressive strength declines associated with UPOFA content. Particularly, the curvature of SF-ST-PET (B, C, and D) was sharper than the convex shape for UPOFA (A). That may indicate that SF-ST-PET variables affected compressive strength at all ages more than UPOFA. Thereby, UPOFA (A) exhibits increasing compressive strength at intermediate values and decreasing it at high levels. In contrast, SF (B), ST (C), and PET (D) show a linear positive correlation, leading to a consistent increase in compressive strength with higher levels. Therefore, Post-hoc tests were not conducted as the principal analysis showed that the compressive strength increased significantly due to a combination of silica, steel fibers, and PET fibers, so the extra post-hoc comparisons were not necessary.

### 3.4 Optimization of multiple responses

**Figure 12.**
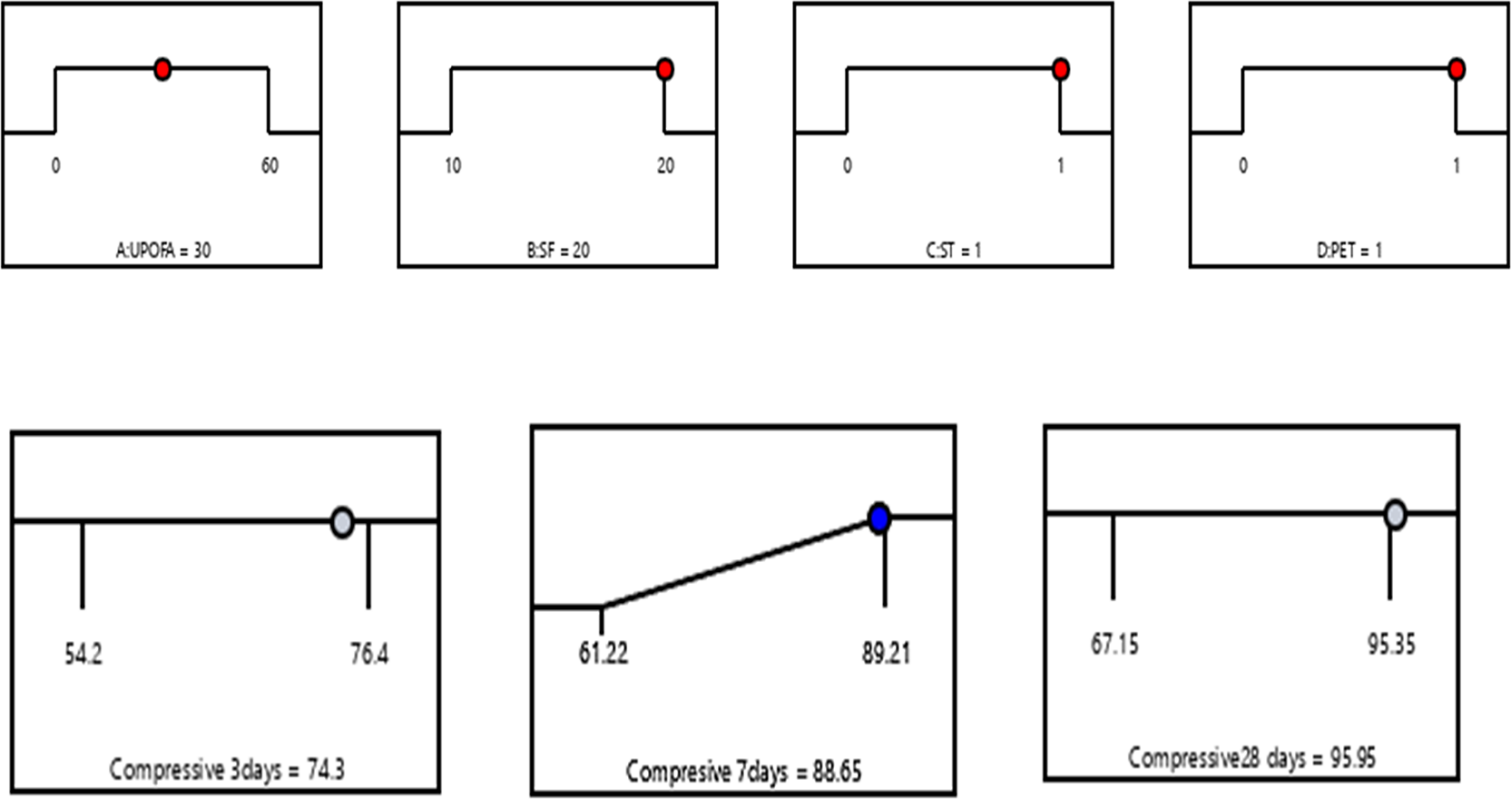
Ramp function graph for optimum compressive strength at 3, 7, and 28 days.

In the numerical optimization process, the factors UPOFA, SF, ST, and PET fiber were assigned specific ranges. At the same time, the compressive strength responses at the ages of 3, 7, and 28 days were also defined within certain limits. The main objective of combining SF, ST, and PET is to achieve the highest compressive strength values at early ages for GHSC containing high amounts of UPOFA (30% and 60%). To identify the optimal conditions, factors based on the Response Surface Methodology (RSM) desirability criterion are utilized. The model equations were solved simultaneously to identify the process variables. At the optimal state, the compressive strengths achieved were 74.3 MPa, 88.65 MPa, and 95.95 MPa at the ages of 3, 7, and 28 days, respectively. This effect was achieved with 30% UPOFA, 20% SF, 1% ST, and 1% PET. As a result, the compressive strength of GHSC was optimized, leading to a desirability value of 0.98 based on the variable factors and responses, as illustrated in the graphical ramp from Figure 12. There is indeed a need to mention that the optimized concrete mix includes silica, steel fiber, and PET fiber, has a much better compressive strength, ductility, and crack resistance. They increase its applicability especially in structural components with high load needs such as beams, columns, and slabs, and in areas where it is important that both durability, impact resistance, and long-term performance. The blending of fibers and pozzolanic substances will offer a sure way of minimizing the maintenance and the service life of the concrete structures.

Further, this study primarily focused on early-age mechanical ability, conducted within a controlled civil engineering laboratory and environment. Consequently, additional future works are required to investigate durability, long-term performance, and comprehensive structural validation.

## 4. Conclusions

This work used RSM to assess the feasibility of hybridizing different materials, including SF, steel, and PET fiber, to improve the significant decrease in compressive strength at early ages in GHSC containing substantial quantities of UPOFA. The following conclusions may be derived from the findings.

(1) The compressive strength of GHSC decreased as the percentage of UPOFA replaced with OPC increased from 30% to 60%. The lowest strength values were recorded at the ages of 3, 7, and 28 days with a 60% UPOFA concentration. The incorporation of hybrid materials namely as SF, steel fiber, and PET fiber enhances the compressive strength of 30% and 60% UPOFA-GHSC. At the ages of 3, 7, and 28 days, the GHSC mix of 30% UPOFA, 20% SF, 1% ST, and 1% PET (M27) demonstrated superior strength of about 76.4 MPa, 89.21 MPa, and 95.35 MPa, respectively. Furthermore, adding hybrid materials to the GHSC with a higher UPOFA of 60% increased compressive strength (M39) by 17%, 25%, and 18% at 3, 7, and 28 days, respectively, when compared to the control mix.
(2) For the GHSC combination M27, which consists of 30% UPOFA, 20% SF, 1% ST, and 1% PET, RSM predicts the optimum conditions. The results of the ANOVA show that SF ST-PET has a greater effect on compressive strength than UPOFA. The experimental findings were well predicted by the quadratic regression, with R ^2^ values of 0.9697, 0.9564, and 0.9674 for compressive strength at 3, 7 and 28 days, respectively. Hence, RSM might be a useful method for improving the mix design of green concrete with UPOFA, which reduces its strength at early ages. By lowering cement production energy consumption and waste volume, this method has the potential to increase the sustainability of concrete, make UPOFA more widely used in the industry, and lessen the negative environmental consequences.

## Data Availability

The datasets supporting the findings of this study are openly available in the Zenodo repository at https://doi.org/10.5281/zenodo.17991317 ^65^ under a Creative Commons Attribution 4.0 International license (CC-BY 4.0).
