# Supplementary material for: Optimizing Early Compressive Strength of Ultrafine POFA-Based Green HSC: An Experimental and RSM Approach with Silica Fume and Hybrid Fibers
Source: F1000Res. 2026 Jun 5;15:103. Originally published 2026 Jan 22. [Version 4] doi: 10.12688/f1000research.175237.4 (PMC13221629; doi:10.12688/f1000research.175237.4)
Supplement: Peer review history

# Reviewer report: Reviewer response for version 3

**DOI:** 10.5256/f1000research.199054.r482869

**Published:** 2026-05-29

**Authors:** Monita Olivia

**Affiliations:** 1 Universitas Riau, Pekanbaru, Indonesia

Please improve the manuscript based on the following comments:

 1) Abstract. The final conclusion focused more on RSM than on the optimal mixes obtained using the method. Please improve this part by stating the optimum results and the comparison with the control mix. 

 2) The research significance must include the research problem statement and the novelty of the study, rather than the research aim. What is the research problem, and what is the novelty of this study that needs to include the RSM in the design method? Why do we need optimization? 

 3) Furthermore, the aim of this research includes tensile strength, but there's no sign of tensile strength data presented in the manuscript. 

 3) Section 3.4, Statement of optimization will result in higher mechanical properties, ductility, crack resistance, etc., must be presented with evidence. How did the authors produce the graphs in Figure 12? Which data contribute to those graphs?

 4) Conclusions. Please provide only conclusions here; e.g., no. 3 is more a recommendation/implication that can be presented after the conclusion points. This manuscript draws some important conclusions based on the research aim (compressive strength at 3, 7, and 28 days, mathematical modeling). 

 5) This RSM and ANOVA were used for optimization of silica, steel fibers, UPOFA, and PET fibers. How could this method produce a response for each material involved in the mixes? Which of the materials has more significant influence than the others? Please add more explanation on this method than other information on this in the discussion and conclusion. What is the impact of having this optimization overall for the UPOFA mix design?

Is the work clearly and accurately presented and does it cite the current literature?

Yes

If applicable, is the statistical analysis and its interpretation appropriate?

I cannot comment. A qualified statistician is required.

Are all the source data underlying the results available to ensure full reproducibility?

Yes

Is the study design appropriate and is the work technically sound?

Yes

Are the conclusions drawn adequately supported by the results?

Partly

Are sufficient details of methods and analysis provided to allow replication by others?

Yes

Reviewer Expertise:

Durability, geopolymer, sustainable waste in concrete

I confirm that I have read this submission and believe that I have an appropriate level of expertise to confirm that it is of an acceptable scientific standard, however I have significant reservations, as outlined above.

# Response

**Published:** 2026-05-30

**Authors:** Aktham Hatem Qasim

**Affiliations:** University of Fallujah, Al-Fallujah, Al Anbar Governorate, Iraq

We would like to thank the reviewer for the valuable and constructive comments. We have carefully addressed all the points raised and provided a point-by-point response below. All corresponding revisions have been incorporated into the revised manuscript.

- **Abstract. The final conclusion focused more on RSM than on the optimal mixes obtained using the method. Please improve this part by stating the optimum results and the comparison with the control mix.**

 
**Action:**

 The experimental and RSM analysis results showed that the GHSC containing 30% UPOFA, 20% SF, 1% ST, and 1% PET fiber yielded optimum strength at 3, 7, and 28 days.

- **The research significance must include the research problem statement and the novelty of the study, rather than the research aim. What is the research problem, and what is the novelty of this study that needs to include the RSM in the design method? Why do we need optimization? **

**Action:**

 Although higher UPOFA content improves sustainability, it may reduce early compressive strength. Therefore, in this study, RSM was employed to optimize the proportions of ultrafine POFA, silica fume, and hybrid fibers to achieve a sustainable and high-performance green HSC. Thus, the effect of coupling steel, PET fibers, and SF on the compressive and tensile strength of GHSC with low cement content was investigated. This mixture includes up to 60% of UPOFA as a replacement binder with cement at the early ages of 3, 7, and 28 days. While UPOFA was employed as a replacement binder at 30% and 60%, SF was substituted at 10% and 20% of the total mass of the cement binder.

- **Furthermore, the aim of this research includes tensile strength, but there's no sign of tensile strength data presented in the manuscript.**

**Action:**

 Thank you for this valuable comment. The reference to tensile strength was intended as a general background consideration regarding the role of hybrid fibers in concrete performance. However, since the present study focuses exclusively on compressive strength, the relevant statements have been revised to ensure consistency with the reported experimental program and objectives.

  

- **Section 3.4, Statement of optimization will result in higher mechanical properties, ductility, crack resistance, etc., must be presented with evidence. How did the authors produce the graphs in Figure 12? Which data contribute to those graphs?**

**Action:**

 Thank you for this valuable comment. The statement has been revised to ensure consistency with the scope of the present study. The discussion is now limited to the experimental and RSM results related to compressive strength.

- **Conclusions. Please provide only conclusions here; e.g., no. 3 is more a recommendation/implication that can be presented after the conclusion points. This manuscript draws some important conclusions based on the research aim (compressive strength at 3, 7, and 28 days, mathematical modeling).**

**Action:**

 Thank you for this comment. The Conclusions section has been revised to include only the main findings related to compressive strength at 3, 7, and 28 days and mathematical modeling. The recommendation/implication has been removed from the conclusions and presented in a separate Future Work section.

- **This RSM and ANOVA were used for optimization of silica, steel fibers, UPOFA, and PET fibers. How could this method produce a response for each material involved in the mixes? Which of the materials has a more significant influence than the others? Please add more explanation on this method than the other information on this in the discussion and conclusion. What is the impact of having this optimization overall for the UPOFA mix design?**

**Action:**

 Thank you for this valuable comment. The objective of this study was not to evaluate the individual effect of each constituent separately, but rather to investigate the synergistic and interaction effects of silica fume, steel fibers, and PET fibers within a high-volume UPOFA-based green concrete matrix. The experimental results indicated that single-material mixtures did not achieve optimum performance, whereas improved behavior was obtained when the materials were combined due to significant interaction effects. The RSM framework, supported by ANOVA analysis, was employed to evaluate the significance of main and interaction effects, develop a predictive model, and reduce the number of experimental trials compared to conventional experimental approaches. This allowed efficient optimization of the mixture composition and identification of the optimum design rather than ranking individual materials. Overall, this demonstrates the effectiveness of the RSM–ANOVA approach in optimizing high-volume UPOFA-based green concrete systems while minimizing experimental effort.

# Reviewer report: Reviewer response for version 2

**DOI:** 10.5256/f1000research.196708.r469919

**Published:** 2026-04-13

**Authors:** Farid Debieb

**Affiliations:** 1 University of Medea, Medea, Algeria

The manuscript presents a well-founded and effectively implemented study. The experiment plans, the use of statistics for data analysis, and the conclusions are all quite fitting. However, it fails in giving enough information for a complete duplication, especially regarding UPOFA processing, the mixing procedure, compaction, and workability data. Besides, there are minor problems with clarity, like repetition and references to figures. Here are some ways that might improve the paper's overall strength and clarity:

 1- Why were steel fiber (ST) and PET fiber only at 0% or 1% levels and no intermediate levels (e.g., 0.5% or 1.5%) were considered, whereas UPOFA and SF each had three levels?

 2 The article states that the compressive strength drops at 60% UPOFA are because of the dilution and low production of C-S-H. However, could you clarify the part of overconsumption of CH due to a high amount of amorphous silica?

 3- According to the ANOVA results (Table 6), nearly all two-way interactions (AB, AC, AD, BC, BD) are not statistically significant (p > 0.05). So, is using a full quadratic RSM model necessary?

 4- The study looks at only the early-age mechanical strength (3, 7, 28 days). What are the most important durability properties that need to be examined before this optimized GHSC can be used on an industrial scale?

 5- Is it possible that the findings of this study apply to other pozzolanic wastes (e.g., rice husk ash) or other synthetic fibers (e.g., polypropylene, PVA)?

Is the work clearly and accurately presented and does it cite the current literature?

Partly

If applicable, is the statistical analysis and its interpretation appropriate?

Yes

Are all the source data underlying the results available to ensure full reproducibility?

Partly

Is the study design appropriate and is the work technically sound?

Yes

Are the conclusions drawn adequately supported by the results?

Yes

Are sufficient details of methods and analysis provided to allow replication by others?

Partly

Reviewer Expertise:

innovative materials for construction, concrete technology, recycled concrete

I confirm that I have read this submission and believe that I have an appropriate level of expertise to confirm that it is of an acceptable scientific standard.

# Response

**Published:** 2026-04-17

**Authors:** Aktham Hatem Qasim

**Affiliations:** University of Fallujah, Al-Fallujah, Al Anbar Governorate, Iraq

**Reviewer 2**

1. Why were steel fiber (ST) and PET fiber only at 0% or 1% levels and no intermediate levels (e.g., 0.5% or 1.5%) were considered, whereas UPOFA and SF each had three levels?

**Action**

**Thanks for the reviewer’s important comment**. The use of steel fiber (ST) and PET fiber was limited to only 0% and 1% without intermediate levels, as previous studies have consistently shown that the most significant effect of these types of fibers—especially when combined with pozzolanic materials—occurs around this percentage. Therefore, selecting 0% and 1% was sufficient to capture the primary influence of fiber addition, while avoiding unnecessary intermediate levels that may not produce substantially different results. In contrast, UPOFA and silica fume (SF) were studied at multiple levels because their influence on material properties is more gradual and requires a broader range to fully evaluate their effect. 

  

1. The article states that the compressive strength drops at 60% UPOFA are because of the dilution and low production of C-S-H. However, could you clarify the part of overconsumption of CH due to a high amount of amorphous silica?

**Action**

**Thanks for the reviewer’s careful reading.** (Done, added to manuscript see subsection 3.1). The increased concentration of amorphous silica, including 60% UPOFA, accelerates the consumption rate of Calcium Hydroxide (CH); nevertheless, the reduced cement content hampers its production in areas where CH is being exhausted. This impact results in the pozzolanic reaction attaining a CH-limited state, thereby leading to a reduced and delayed synthesis of Calcium Silicate Hydrate (C-S-H). Thereby, the interaction between the availability of calcium hydroxide and silica content in palm oil fuel ash significantly influences the evolution of compressive strength.

1. According to the ANOVA results (Table 6), nearly all two-way interactions (AB, AC, AD, BC, BD) are not statistically significant (p > 0.05). So, is using a full quadratic RSM model necessary?

**Action**

**Thanks for the reviewer’s careful reading.** Although most of the two-way interactions were statistically insignificant (p > 0.05), the entire quadratic RSM model was retained to retain hierarchy and explain any curvature. The high statistical measures (R2> 0.93 and Precision> 24) also affirm the sufficiency of the model and a high predictive power and outstanding signal to noise.

1.  The study looks at only the early-age mechanical strength (3, 7, 28 days). What are the most important durability properties that need to be examined before this optimized GHSC can be used on an industrial scale?

**Action**

1. **Thanks for the reviewer’s important comment. (Noted, done, the future work paragraph has been added at the end of the conclusion section).** Future works are required in the optimization of GHSC by RSM in terms of long-term durability performance in diverse environmental factors, such as chloride penetration, sulfate attack, carbonation, water absorption, and permeability. Moreover, machine learning in combination with RSM can make its predictions more accurate and create a more resilient multi-objective optimization model that will consider the mechanical and durability characteristics of sustainable concrete design.
2.  Is it possible that the findings of this study apply to other pozzolanic wastes (e.g., rice husk ash) or other synthetic fibers (e.g., polypropylene, PVA)?

**Action**

**Thanks for the reviewer’s important comment. **Other pozzolanic fibers such as Rice Husk Ash and synthetic fibers, such as Polypropylene Fiber and Polyvinyl Alcohol Fiber can be qualitatively checked using the results since they are pozzolanically similar and react with the fiber-pozzolana mechanisms. It is however a system dependent utility since the variations in chemical compositions, curing, particle and interfacial properties can play a major role and thus should be assured on a case by case analysis.

# Reviewer report: Reviewer response for version 1

**DOI:** 10.5256/f1000research.193200.r456473

**Published:** 2026-02-11

**Authors:** Sattawat Haruehansapong

**Affiliations:** 1 Rajamangala University of Technology Tawan-ok, Bangkok, Thailand

The manuscript presents a well-structured experimental and modeling study on the optimization of early-age compressive strength of ultrafine POFA-based green high-strength concrete. The experimental program is sound, and the statistical modeling approach is appropriate. The conclusions are generally supported by the presented evidence.

 However, several **minor but important revisions** are recommended to improve clarity, conciseness, and engineering relevance before final approval.

 1. Title

 The title is informative but could better reflect the use of statistical optimization techniques.

**Suggested action: **Consider explicitly mentioning the use of response surface methodology or statistical optimization.

 2. Abstract

- Clearly explain why early-age compressive strength is critical, particularly for UPOFA-based concrete.
- Explicitly state the application scope (non-structural or low-load applications).
- Separate experimental findings from modeling outcomes more clearly.
- **Suggested action:**
- Add one sentence clarifying application limitations.
- Revise for conciseness.

3. Introduction

1. Reduce repetition related to general sustainability concepts.
2. Strengthen the research gap, particularly regarding hybrid fiber systems in high-volume UPOFA concrete.
3. Clearly distinguish this study from previous related works.

4.1 Materials Characterization

- Consider adding a summary table describing the role and expected effect of each material (UPOFA, silica fume, steel fiber, PET fiber).

4.2 Experimental Design and RSM

1. Justify why steel fiber and PET fiber contents were fixed at specific values.
2. Briefly justify the selection of CCD over other RSM designs.

5.1 Compressive Strength Results

1. Reduce narrative repetition of numerical values already presented in figures.
2. Strengthen microstructural explanations linking UPOFA dilution, silica fume nucleation, and fiber bridging effects.

5.2 Statistical Analysis and Modeling

1. Briefly explain why post-hoc statistical tests were not conducted.
2. Clearly distinguish statistical significance from engineering significance.

6. Optimization Results and Practical Implications

- Add a short subsection explicitly addressing engineering applications of the optimized mix.

**Suggested action:**

- Clearly state where and how the optimized concrete can be practically used.

7. Conclusions

1. Avoid repeating detailed numerical results.
2. Explicitly acknowledge study limitations (laboratory scale, early-age focus).
3. Provide a clear direction for future work.

Is the work clearly and accurately presented and does it cite the current literature?

Partly

If applicable, is the statistical analysis and its interpretation appropriate?

Partly

Are all the source data underlying the results available to ensure full reproducibility?

Partly

Is the study design appropriate and is the work technically sound?

Yes

Are the conclusions drawn adequately supported by the results?

Yes

Are sufficient details of methods and analysis provided to allow replication by others?

Yes

Reviewer Expertise:

concrete, materials, cement

I confirm that I have read this submission and believe that I have an appropriate level of expertise to confirm that it is of an acceptable scientific standard, however I have significant reservations, as outlined above.

# Response

**Published:** 2026-02-16

**Authors:** Aktham Hatem Qasim

**Affiliations:** University of Fallujah, Al-Fallujah, Al Anbar Governorate, Iraq

**Response to Reviewer Comments**

 The authors thank the reviewer for their careful reading and constructive comments below. We are very pleased to receive the reviewer’s recognition of the research work in this paper. We believe that we have addressed all of the comments through our responses below, as well as in the text of the manuscript when appropriate.

 1. Title

 The title is informative but could better reflect the use of statistical optimization techniques.

 Suggested action: Consider explicitly mentioning the use of response surface methodology or statistical optimization.

**Action**

   Thanks for the reviewer’s important comment. (Noted, done)

 2. Abstract

 Clearly explain why early-age compressive strength is critical, particularly for UPOFA-based concrete.

 Explicitly state the application scope (non-structural or low-load applications).

**Action**

   Thanks for the reviewer’s important comment. (Noted, done)

 Moreover, the replacement rates of HSGC are probably high in POFA, which is constrained in the areas of the application where the removal of the formwork or pre-stressing of the structures is required in a very short period of time, and the high early-age strengths are required. ^16^

 Separate experimental findings from modeling outcomes more clearly.

 Suggested action:

**Action**

**Thanks for the reviewer’s suggestion, as shown, experimental findings and modeling outcomes are presented separately.**

 Add one sentence clarifying application limitations.

**Action**

****Thanks for the reviewer’s important comment (Noted, done). However, somehow these studies suggested the limitation of the POFA used because the highest was limited. The compressive strength was determined at a POFA replacement level of 20%, and the ultimate highest compressive strength of 28-days compressive strength achieved with the concretes that used POFA ranged between 60 and 86 MPa **. ^17, 18^**

 3. Introduction

 Reduce repetition related to general sustainability concepts.

**Action**

**Thanks for the reviewer’s comment (Noted, done, within introduction part).**

 Strengthen the research gap, particularly regarding hybrid fiber systems in high-volume UPOFA concrete.

**Action**

**Thanks for the reviewer’s comment (Noted, the research significance subsection already addresses the gap regarding hybrid fiber systems in high-volume UPOFA concrete.**

 Clearly distinguish this study from previous related works.

**Action**

**Thanks for the reviewer’s comment (Noted, the Introduction already highlights the study’s novelty and clearly distinguishes it from previous works.**

 4.1 Materials Characterization

 Consider adding a summary table describing the role and expected effect of each material (UPOFA, silica fume, steel fiber, PET fiber).

**Action**

**Thanks for the reviewer’s excellent suggestion. (Noted, done, see Table 2).**

 4.2 Experimental Design and RSM

 Justify why the steel fiber and PET fiber contents were fixed at specific values.

**Action**

 Thanks for the reviewer’s careful reading. (Noted, done). The contents of the steel and PET fibers were set to 1% volume, which is consistent with previous studies reporting that this amount has improved mechanical performance, crack control, and ductility whilst maintaining workability ^.21, 36^

 Briefly justify the selection of CCD over other RSM designs.

**Action**

**Thanks for the reviewer’s comment. (Noted, done). **CCD technique is favored to the full factorial and three-level design as it estimates the linear, quadratic and interaction effects with minimum tests, which will lead to a good model fitting, greater accuracy in prediction, reduced amount of residual error, and a high value of R2, that could permit the creation of three-dimensional response surfaces with great reliability. Consequently, it has commonly been considered as the best experimental design to investigate the second-order response models. ^4, 15^

 5.1 Compressive Strength Results

 Reduce narrative repetition of numerical values already presented in figures.

 Thanks for the reviewer’s comment. Due to the high number of mixes and material changes, numerical values are supplied in the text in addition to tables and figures to assist the reader in understanding and following the patterns in the results.

 Strengthen microstructural explanations linking UPOFA dilution, silica fume nucleation, and fiber bridging effects.

**Action**

**Thanks for the reviewer’s careful reading. (Noted, done)**

 The compressive strength of GHSC is enhanced over a curing period of 7 to 28 days as a result of the accelerated hydration of conventional OPC and due to the pozzolanic effects of SF. The formation of secondary C-S-H is the consequence of the interaction between (Ca(OH)₂) produced during cement hydration and SiO₂ in SF ^61,62^. This procedure increases the durability of the concrete by reducing the incidence of microcracks during periods of tension. In the same trend, adding steel and PET fibers improved the compressive strength of HSC and GHSC. The GHSC mixtures with a combination of (UPOFA-SF-ST-PET) registered the highest compressive strength of mixtures in GII and GIII at all curing periods. Hereby, the compressive strength of (M39) GHSC-U60 increased by 17%,25% and 18% at ages of 3, 7, and 28 days, respectively, related to control mix. This strength development was attributed to the effectiveness of binary binders (UPOFA and SF) in increasing bond formation between steel and PET fibers and other concrete ingredients.  The tiny particle size and pozzolanic reactivity of UPOFA and SF reduced void volume and produced extra C-S-H gels, strengthening the aggregate-paste interfacial connection and increasing bonding capabilities with fibers. Adding steel and PET fibers may improve the fracture resistance of fiber-reinforced GHSC composites by limiting micro-crack formation under load, which is accomplished by forming a bridge network within the concrete microstructure ^33,34,64^

 5.2 Statistical Analysis and Modeling

 Briefly explain why post-hoc statistical tests were not conducted.

**Action**

**Thanks for the reviewer’s important comment. (Noted, done).** Therefore, Post-hoc tests were not conducted as the principal analysis showed that the compressive strength increased significantly due to a combination of silica, steel fibers, and PET fibers, so the extra post-hoc comparisons were not necessary.

 .Clearly distinguish statistical significance from engineering significance.

**Action**

**Thanks for the reviewer’s comment. (Noted).**

 The practical relevance of these variations on material performance or structural design is reflected in engineering significance, whereas statistical significance shows that observed differences are unlikely to be the result of chance (e.g., p<0.05). Despite having little engineering significance, certain outcomes could be statistically significant.

 6. Optimization Results and Practical Implications

 Add a short subsection explicitly addressing engineering applications of the optimized mix.

**Action**

**Thanks for the reviewer’s important comment. (Noted, done).** There is indeed a need to mention that the optimized concrete mix includes silica, steel fiber, and PET fiber, has a much better compressive strength, ductility, and crack resistance. They increase its applicability especially in structural components with high load needs such as beams, columns, and slabs, and in areas where it is important that both durability, impact resistance, and long-term performance. The blending of fibers and pozzolanic substances will offer a sure way of minimizing the maintenance and the service life of the concrete structures.

 Suggested action:

 Clearly state where and how the optimized concrete can be practically used.

**Thanks for the reviewer’s suggestion.**

 7. Conclusions

 Avoid repeating detailed numerical results.

 Explicitly acknowledge study limitations (laboratory scale, early-age focus).

 Provide a clear direction for future work  

**Action**

**Thanks for the reviewer’s important comment. (Noted, done).** Further, this study primarily focused on early-age mechanical ability, conducted within a controlled civil engineering laboratory and environment. Consequently, additional future works are required to investigate durability, long-term performance, and comprehensive structural validation.

# Response

**Published:** 2026-04-17

**Authors:** Aktham Hatem Qasim

**Affiliations:** University of Fallujah, Al-Fallujah, Al Anbar Governorate, Iraq

**Reviewer 1**

 1. Title

 The title is informative but could better reflect the use of statistical optimization techniques.

 Suggested action: Consider explicitly mentioning the use of response surface methodology or statistical optimization.

**Action**

   Thanks for the reviewer’s important comment. (Noted, done). **“**Optimizing Early Compressive Strength of Ultrafine POFA-Based Green HSC: An Experimental and RSM Approach with Silica Fume and Hybrid Fibers **”**

 2. Abstract

 Clearly explain why early-age compressive strength is critical, particularly for UPOFA-based concrete.

 Explicitly state the application scope (non-structural or low-load applications).

**Action**

   Thanks for the reviewer’s important comment. (Noted, done)

 Moreover, the replacement rates of HSGC are probably high in POFA, which is constrained in the areas of the application where the removal of the formwork or pre-stressing of the structures is required in a very short period of time, and the high early-age strengths are required. ^16^

 Separate experimental findings from modeling outcomes more clearly.

 Suggested action:

**Action**

**Thanks for the reviewer’s suggestion, **as shown, experimental findings and modeling outcomes are presented separately. (See, subsection 3.2: Mathematical modeling and statistical analysis results.

****

 Add one sentence clarifying application limitations.

**Action**

****Thanks for the reviewer’s important comment (Noted, done). However, somehow these studies suggested the limitation of the POFA used because the highest was limited. The compressive strength was determined at a POFA replacement level of 20%, and the ultimate highest compressive strength of 28-days compressive strength achieved with the concretes that used POFA ranged between 60 and 86 MPa **. ^17, 18^**

 3. Introduction

 Reduce repetition related to general sustainability concepts.

**Action**

**Thanks for the reviewer’s comment (Noted, **the manuscript has been revised to reduce repetition related to general sustainability concepts, particularly in the Introduction section.)

 Strengthen the research gap, particularly regarding hybrid fiber systems in high-volume UPOFA concrete.

**Action**

**Thanks for the reviewer’s comment (Noted, done. See subsection 1.1:  research significance which already addresses the gap regarding hybrid fiber systems in high-volume UPOFA concrete.)**

 Clearly distinguish this study from previous related works.

**Action**

**Thanks for the reviewer’s comment (Noted, **the Introduction has been revised to more clearly highlight the novelty of this study and distinguish it from previous related work.)

 4.1 Materials Characterization

 Consider adding a summary table describing the role and expected effect of each material (UPOFA, silica fume, steel fiber, PET fiber).

**Action**

**Thanks for the reviewer’s excellent suggestion. (Noted, done. **A summary table has been added (Table 2) to clearly describe the role and expected effect of each material, including UPOFA, silica fume, steel fiber, and PET fiber.).

 4.2 Experimental Design and RSM

 Justify why the steel fiber and PET fiber contents were fixed at specific values.

**Action**

 Thanks for the reviewer’s careful reading. (Noted, done). The contents of the steel and PET fibers were set to 1% volume, which is consistent with previous studies reporting that this amount has improved mechanical performance, crack control, and ductility whilst maintaining workability ^.21, 36.  ^Further ^,^ studies done before have indicated that the strongest effect of these types of fibers, especially when combined with pozzolanic substances, is at this ratio. In turn, selecting 0% and 1% was sufficient to capture the main effect of the fiber addition and avoid irrelevant intermediate levels, which would not yield much different results.

 Briefly justify the selection of CCD over other RSM designs.

**Action**

**Thanks for the reviewer’s comment. (Noted, done). **CCD technique is favored to the full factorial and three-level design as it estimates the linear, quadratic and interaction effects with minimum tests, which will lead to a good model fitting, greater accuracy in prediction, reduced amount of residual error, and a high value of R ^2^, that could permit the creation of three-dimensional response surfaces with great reliability. Consequently, it has commonly been considered as the best experimental design to investigate the second-order response models. ^4, 15^

 5.1 Compressive Strength Results

 Reduce narrative repetition of numerical values already presented in figures.

 Thanks for the reviewer’s comment. Due to the high number of mixes and material changes, numerical values are supplied in the text in addition to tables and figures to assist the reader in understanding and following the patterns in the results.

 Strengthen microstructural explanations linking UPOFA dilution, silica fume nucleation, and fiber bridging effects.

**Action**

**Thanks for the reviewer’s careful reading. (Noted, done)**

 The compressive strength of GHSC is enhanced over a curing period of 7 to 28 days as a result of the accelerated hydration of conventional OPC and due to the pozzolanic effects of SF. The formation of secondary C-S-H is the consequence of the interaction between (Ca(OH)₂) produced during cement hydration and SiO₂ in SF ^61,62^. This procedure increases the durability of the concrete by reducing the incidence of microcracks during periods of tension. In the same trend, adding steel and PET fibers improved the compressive strength of HSC and GHSC. The GHSC mixtures with a combination of (UPOFA-SF-ST-PET) registered the highest compressive strength of mixtures in GII and GIII at all curing periods. Hereby, the compressive strength of (M39) GHSC-U60 increased by 17%,25% and 18% at ages of 3, 7, and 28 days, respectively, related to control mix. This strength development was attributed to the effectiveness of binary binders (UPOFA and SF) in increasing bond formation between steel and PET fibers and other concrete ingredients.  The tiny particle size and pozzolanic reactivity of UPOFA and SF reduced void volume and produced extra C-S-H gels, strengthening the aggregate-paste interfacial connection and increasing bonding capabilities with fibers. Adding steel and PET fibers may improve the fracture resistance of fiber-reinforced GHSC composites by limiting micro-crack formation under load, which is accomplished by forming a bridge network within the concrete microstructure ^33,34,64^

 5.2 Statistical Analysis and Modeling

 Briefly explain why post-hoc statistical tests were not conducted.

**Action**

**Thanks for the reviewer’s important comment. (Noted, done).** Therefore, Post-hoc tests were not conducted as the principal analysis showed that the compressive strength increased significantly due to a combination of silica, steel fibers, and PET fibers, so the extra post-hoc comparisons were not necessary.

 .Clearly distinguish statistical significance from engineering significance.

**Action**

**Thanks for the reviewer’s comment. (Noted).**

 The practical relevance of these variations on material performance or structural design is reflected in engineering significance, whereas statistical significance shows that observed differences are unlikely to be the result of chance (e.g., p<0.05). Despite having little engineering significance, certain outcomes could be statistically significant.

 6. Optimization Results and Practical Implications

 Add a short subsection explicitly addressing engineering applications of the optimized mix.

**Action**

**Thanks for the reviewer’s important comment. (Noted, done).** There is indeed a need to mention that the optimized concrete mix includes silica, steel fiber, and PET fiber, has a much better compressive strength, ductility, and crack resistance. They increase its applicability especially in structural components with high load needs such as beams, columns, and slabs, and in areas where it is important that both durability, impact resistance, and long-term performance. The blending of fibers and pozzolanic substances will offer a sure way of minimizing the maintenance and the service life of the concrete structures.

 Suggested action:

 Clearly state where and how the optimized concrete can be practically used.

**Action**

**Thanks for the reviewer’s suggestion.  **( **See subsection 3.4)**

****There is indeed a need to mention that the optimized concrete mix includes silica, steel fiber, and PET fiber, has a much better compressive strength, ductility, and crack resistance. They increase its applicability especially in structural components with high load needs such as beams, columns, and slabs, and in areas where it is important that both durability, impact resistance, and long-term performance. Thus, related to current study the optimized GHSC is applicable in structural elements, which include beams, columns, and precast elements, especially in settings that demand high strength and durability.

 7. Conclusions

 Avoid repeating detailed numerical results.

 Explicitly acknowledge study limitations (laboratory scale, early-age focus).

 Provide a clear direction for future work  

**Action**

**Thanks for the reviewer’s important comment. (Noted, done, the future work paragraph has been added at the end of the Conclusion section).** Future works are required in the optimization of GHSC by RSM in terms of long-term durability performance in diverse environmental factors, such as chloride penetration, sulfate attack, carbonation, water absorption, and permeability. Moreover, machine learning in combination with RSM can make its predictions more accurate and create a more resilient multi-objective optimization model that will consider the mechanical and durability characteristics of sustainable concrete design.
